# Therapeutic siRNA targeting endothelial KDR decreases portosystemic collateralization in portal hypertension

**DOI:** 10.1038/s41598-017-14818-7

**Published:** 2017-11-01

**Authors:** Javier Gallego, Ester Garcia-Pras, Marc Mejias, Nuria Pell, Ute Schaeper, Mercedes Fernandez

**Affiliations:** 1 IDIBAPS Biomedical Research Institute, Hospital Clinic, University of Barcelona, 08036 Barcelona, Spain; 2 CIBER of Hepatic and Digestive Diseases (CIBEREHD), Instituto de Salud Carlos III, Madrid, Spain; 3 SilenceTherapeutics GmbH, Robert Rössle Str. 10, 13125 Berlin, Germany

## Abstract

Development of portosystemic collateral vessels and gastroesophageal varices is responsible for the most serious clinical consequences of portal hypertension, but effective clinical therapies are limited. Here we developed and investigated the therapeutic potential of an innovative liposomally-formulated short-interfering RNA (siRNA) technology based on clinical stage components, capable to attenuate production of the endothelial kinase insert domain receptor (KDR), which controls portosystemic collateralization and contributes to disease progression and aggravation. These siRNAs were first validated *in vitro*, and then, their therapeutic potential on portosystemic collateralization and pathological angiogenesis was tested *in vivo* in mouse models of portal hypertension (portal vein-ligation). siRNA^KDR^-lipoplexes efficiently transported siRNA^KDR^ to vascular endothelial cells in mesenteric microvenules and portal vein of portal hypertensive mice, where collaterogenesis and angiogenesis take place. This systemic treatment significantly downregulated pathological KDR overexpression, without causing complete KDR knockout, preserving homeostatic baseline KDR levels and thus limiting adverse effects. siRNA^KDR^-lipoplex-induced endothelial-specific KDR knockdown drastically reduced by 73% the portosystemic collateralization, and impaired the pathologic angiogenic potential of vascular endothelial cells at different levels (cell proliferation, sprouting and remodeling). Targeting endothelial KDR with therapeutic siRNA^KDR^-lipoplexes could be a promising and plausible treatment modality for attenuating the formation of portosystemic collaterals in a clinical setting.

## Introduction

Portal hypertension is one of the most significant complications of chronic liver diseases, which represent serious threats to human health^1,2^. A major determinant of the severity of portal hypertension and liver disease is the development and maintenance of portosystemic collateral vessels, which include the gastroesophageal varices^3–5^. These varices are fragile and particularly prone to leak blood and even rupture, causing upper gastrointestinal tract bleeding. This hemorrhage is often torrential and difficult to staunch, and, despite many advances made in this field, it continues to be the cause of significant morbidity and mortality in patients^3–5^. Furthermore, because portosystemic collaterals shunt blood from the portal vein to the systemic circulation bypassing the liver, noxious substances that are normally metabolized by the liver, such as drugs, toxins, hormones and bacteria, can escape to the central venous system, leading to other potentially lethal consequences, such as portosystemic encephalopathy, spontaneous bacterial peritonitis or systemic infections. Therefore, one major objective in the treatment of portal hypertension and liver disease in humans is the prevention and reduction of portosystemic collateral growth^3–5^.

Portosystemic collaterogenesis in portal hypertension and chronic liver disease is currently being recognized as a complex dynamic process that involves the reopening of collapsed embryonic channels, and the de novo formation of new collateral vessels through sprouting angiogenesis^6–15^. Newly formed collaterals undergo additional structural and functional changes through vascular remodeling^6–15^. Kinase insert domain receptor (KDR), also known as VEGF receptor-2^16–20^, is an endothelial cell surface receptor that is abundantly expressed in portal hypertension but not in normal tissues, and has a central role in the regulation of portosystemic collateral development^6–10^, and pathological angiogenesis^16–20^, as we and other groups have previously demonstrated. This means that KDR could be an excellent therapeutic target for portal hypertension and chronic liver disease^21–23^. This is especially important because, although the pathophysiology and clinical consequences of portosystemic collateral growth have been studied in detail, this knowledge has not been accompanied by parallel advances in therapies.

Here we describe a new therapeutic strategy for treatment of portosystemic collateralization in portal hypertension. This treatment is based on short interfering RNA (siRNA) technology^24–26^, which has broad potential as a therapeutic to attenuate production of specific target proteins *in vivo* and for the treatment of disease. It has in fact emerged as one of the most promising platforms for therapeutic product development. In particular, we have developed highly effective siRNA sequences against KDR (siRNA^KDR^), which have been chemically modified to provide stability in the bloodstream and evasion of the immune system. These siRNA^KDR^ have also been encapsulated within clinically suitable delivery materials to form siRNA^KDR^-lipoplexes, which not only preserve biological activity but also enhance stability and systemic delivery specifically to vascular endothelial cells^27–32^. Our results demonstrate the efficacy of this improved treatment modality to specifically knockdown the disease-induced KDR overexpression, and robustly attenuate the severity of portosystemic collateral vessels in a murine model of portal hypertension. Of interest, a related type of formulation that we have also developed has been shown to be viable and well tolerated for systemic siRNA administration and is currently being evaluated in clinical trials for treatment of patients with advanced solid tumors^31^, further supporting the translational relevance and therapeutic potential of this approach for portal hypertension and chronic liver disease in a clinical context. Given the emerging roles of angiogenesis in a number of human pathologies, including inflammation, obesity and tumor growth, siRNA^KDR^-lipoplexes may provide a novel strategy to treat a wide spectrum of diseases.

## Methods

Additional methods described in online supplementary information include: Immunoblotting, and RNA isolation and real-time reverse transcriptase (RT)-PCR analysis.

### *In vitro* siRNA transfection

**Figure 1:**
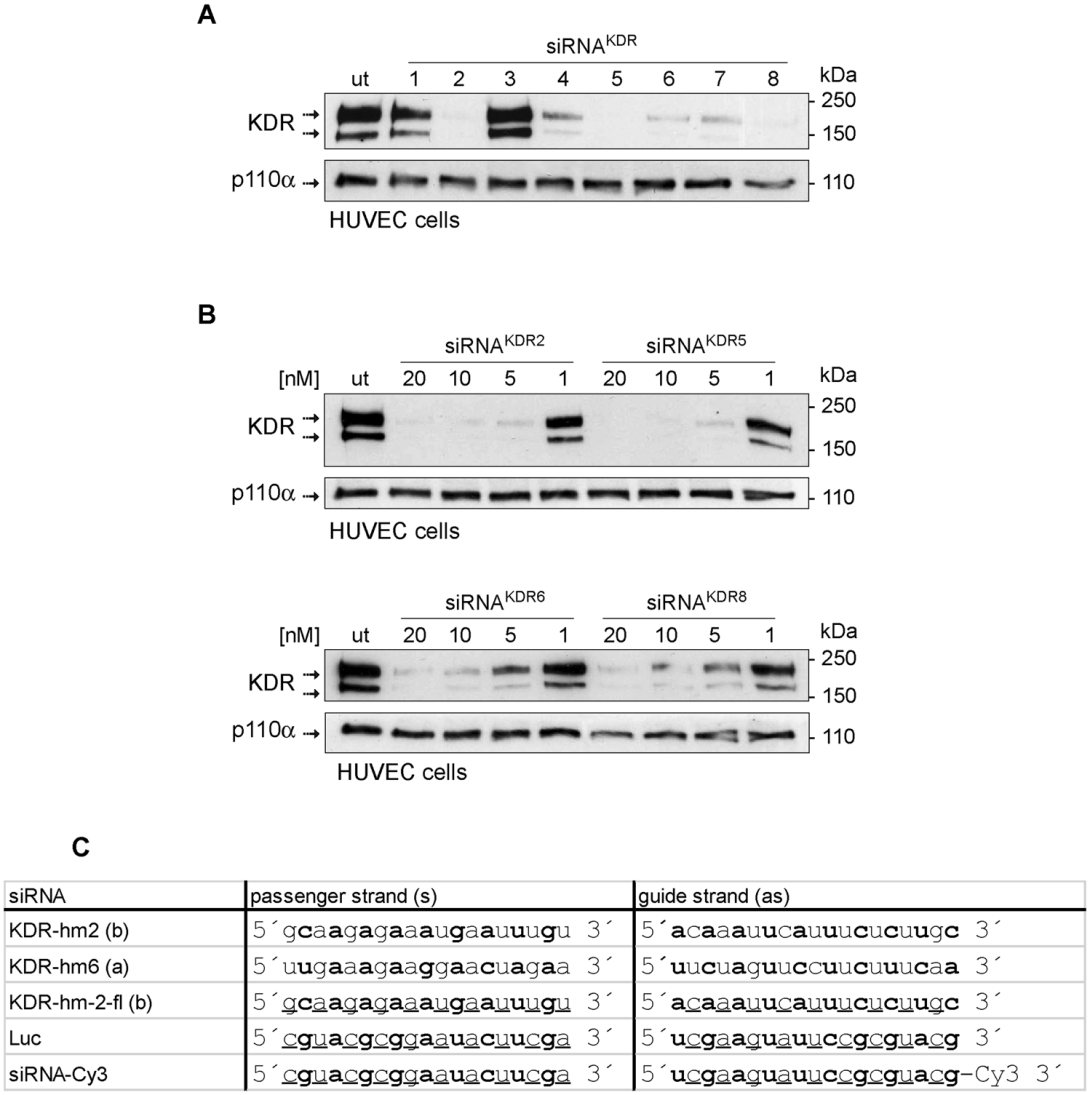
*In vitro* test of in silico selected siRNAs molecules for targeting KDR. (**A**) Human umbilical vein endothelial cells (HUVECS) were seeded in 6 well dishes at a density of 50,000 cells/well and transfected with 20 nM siRNA and 1 µg/mL AtuFECT as indicated. Two days after transfection cells were lysed and KDR expression was evaluated by immunoblotting analysis with KDR antibodies. Several siRNAs reduced expression of KDR. Expression of the house keeping gene p110α was not affected the siRNA transfections and was used as loading control. **(B)** Dose-dependent inhibition of KDR protein expression by selected KDR siRNAs. HUVECS were transfected with 20, 10, 5 and 1 nM of the selected siRNAs and AtuFECT at a constant siRNA to lipid ratio of 20 nM siRNA and 1 µg/mL lipid. Strongest reduction of KDR expression was observed by treatment with siRNA^KDR2^. **(C)** siRNA sequences used in this study. Nucleotides with 2′Ome modification are depicted in bold letters, nucleotides with 2′-fluoro modification are underlined. Whole blots are shown in Supplementary Fig. S1.

siRNAs targeting human and mouse KDR were identified by in silico selection based on siRNA algorithm developed at Silence Therapeutics and functional *in vitro* tests initially performed in human umbilical vein endothelial cells (HUVECs) (Fig. 1A and B; full blots are shown in Supplementary Fig. S1). siRNAs that were used in this study are listed in Fig. 1C. They were synthesized by Biospring/Frankfurt a.M. Further *in vitro* siRNA transfection experiments were done in the murine endothelioma H5V endothelial cell line^33–35^. H5V cells were seeded on 6-well plates in 10% DMEM medium containing 10% fetal bovine serum, and incubated at 37 °C in a 5% CO_2_ humidified incubator. When cultures reached 60% confluence, gene-specific siRNA-lipoplexes were prepared by mixing siRNA and lipids (AtuFECT^TM^) and added to cells at a final concentration of 80 nM. Each transfection was performed in triplicate. Two different sequences of siRNA^KDR^ were used (siRNA^KDRa^ and siRNA^KDRb^; Fig. 1C). Empty liposome without siRNA and unrelated luciferase siRNA (siRNA^Luc^) were used as controls to distinguish sequence-specific silencing from non-specific effects. In particular, the siRNA^Luc^ control is designed to have no known target in the cells being used (obviously the firefly luciferase gene is not present in mouse cells). Following incubation at 37 °C for 4 h, the culture medium was replaced with 2 mL of fresh medium supplemented with 10% fetal bovine serum. Cells were cultured under standard conditions for a further 48 h before being examined by real time PCR and immunoblotting to assess efficacy of gene silencing.

### *In vitro* Matrigel tube formation assay

To analyze capillary tube-formation, ice-cold Matrigel (300 μL/well; BD Corning) was poured into 24-well culture plates and allowed to gel at 37 °C for 1 h. After 72 h of transfection, H5V cells (100,000 cells/well) were plated in fresh medium (DMEM supplemented with 10% FBS, 2 mM L-glutamine, 1% penicillin/streptomycin) and incubated for 16 h at 37 °C and 5% CO_2_. Then, cells were examined by phase-contrast microscopy and photographed at 25X and 50X. Tube formation was evaluated and scored as previously described^34^: (0) individual cells, well-separated; (1) cells begin to migrate and align themselves; (2) capillary tubes visible, no sprouting; (3) sprouting of new capillary tubes visible; (4) closed polygons begin to form; (5) complex mesh-like structures develop. Several random view-fields (4–8) per well were examined and the values averaged.

### Animal model of portal hypertension

Portal hypertension was induced in male BALB/C mice weighing 25–30 g (Charles River) by partial portal vein ligation (PPVL)^7,34^. Under isofluorane anesthesia, a midline abdominal incision was made. The portal vein was separated from surrounding tissue, and a calibrated constriction was performed using a single ligature of 5–0 silk tied around the portal vein and a blunt-tipped 27-gauge needle. The needle was then removed, leaving a calibrated constriction of the portal vein. In sham-operated control mice, the portal vein was similarly manipulated but not ligated. All animals’ studies were approved by the Laboratory Animal Care and Use Committees of the University of Barcelona, and were complied with the National Institute of Health (NIH) guidelines on handling of experimental animals.

### Intravenous administration of siRNA-lipoplexes

**Figure 2:**
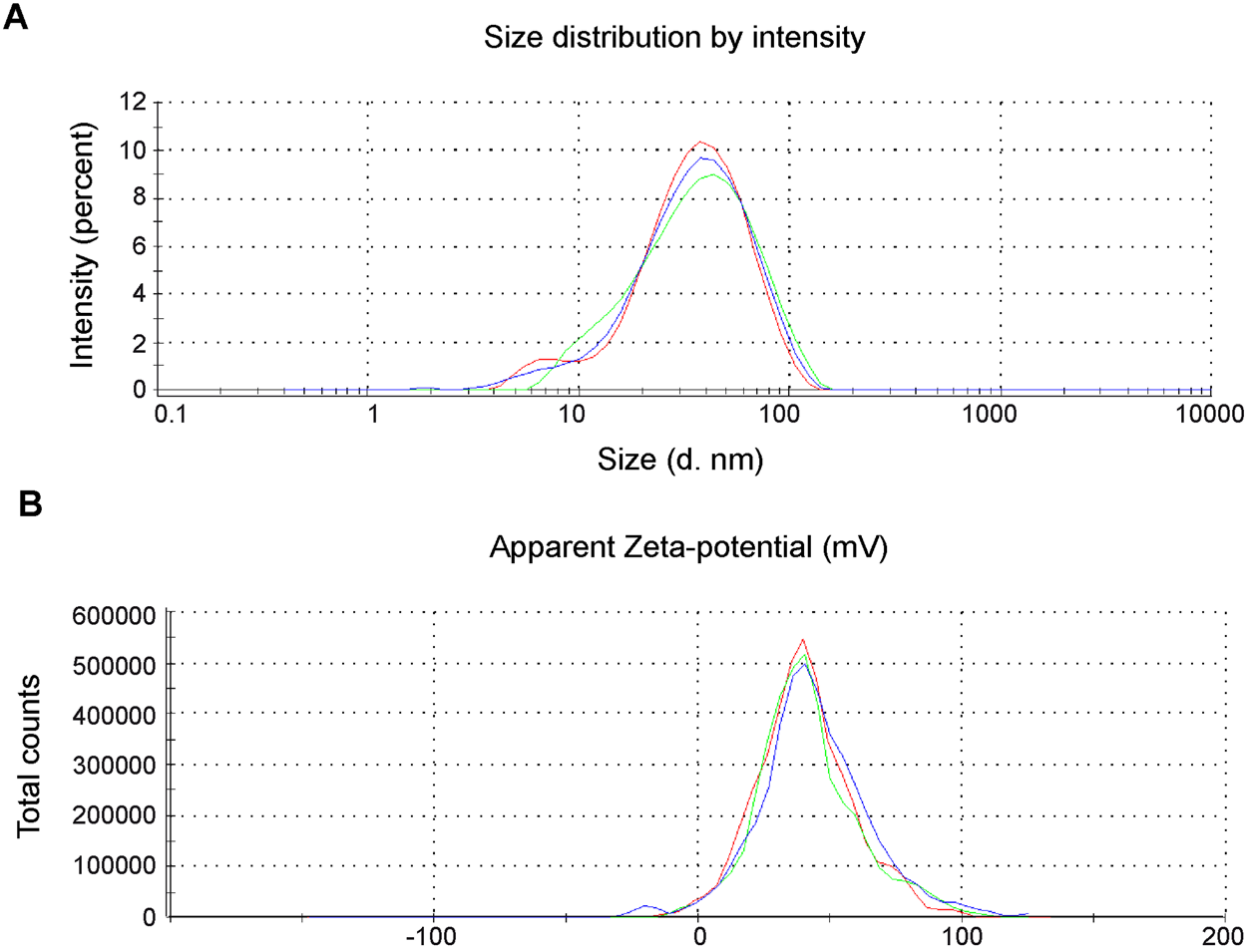
Physico-chemical characterization of AtuPLEX2. AtuPLEX2 lipoplex is composed of 70 mol% cationic lipid AtuFECT01, 1 mol% CerC8 PEG, 29% cholesterol and blunt ended siRNA duplexes with alternating modifications on both strands. **(A)** Size distribution:Z-average size of 29 nm as determined by dynamic light scattering (intensity distribution), measured in 270 mmol/L sucrose buffer, in triplicates. **(B)** The Zeta potential of representative lipoplexes has iis peak at 42.1 mV, measured in 270 mmol/L sucrose buffer, in triplicates.

For *in vivo* studies, we used AtuPLEX2, a derivative of our previously described and characterized siRNA-lipoplex formulation AtuPLEX^TM^ ^27,36^. Briefly, AtuPLEX2 is composed of the cationic lipid system AtuFECT01 (β-L-arginyl-2,3-L-diaminopropionic acid-N-palmityl-N-oleyl-amide trihydrochloride), cholesterol and ceramide C8 Peg (N-octanoyl-sphingosine-1-{succinyl[methoxy(polyethylene glycol)2000]}) in a molar ratio of 70:29:1, and blunt ended siRNA duplexes chemically stabilized by alternating 2′-O-methyl, 2′-fluoro modification on both strands (Fig. 1C). The formulation was prepared in 270 mmol/L sucrose. AtuPLEX2 particles display a Z-average size of 30-nm with a polydispersity index of 0.273 as determined by dynamic light scattering (Fig. 2A and B). AtuPLEX2 prepared with KDR-targeted siRNAs (siRNA^KDR^-lipoplexes) was intravenously injected into the tail vein of male BALB/c mice (n = 15), as described before^28^. AtuPLEX2 prepared with unrelated luciferase siRNAs (siRNA^Luc^-lipoplexes) was injected in control mice (n = 17). siRNA-lipoplex solution contained 0.28 mg/mL siRNA and 2.37 mg/mL lipid (equivalent to a dose of 2.8 mg/kg siRNA and 23.7 mg/kg lipid). Injections were given at a low volume of 300 μL/30 g mouse, using a 1-mL syringe attached to a 27 G needle. siRNA-lipoplexes were diluted in 270 mmol/L sucrose to keep the administration volume of 300 μL/30 g mouse constant. For visualization of cellular uptake *in vivo*, portal hypertensive mice were treated with a single tail vein injection of AtuPLEX2 prepared with siRNA molecules labeled with the fluorophore Cy3 (2.8 mg/kg)^27,28^. Organs were harvested 60-min later, and fluorescence patterns were analyzed in paraffined tissue sections by high-resolution confocal fluorescence microscopy. For determination of the silencing capability of siRNA^KDR^-lipoplexes *in vivo*, in mice with portal hypertension, animals were treated with four tail-vein injections of siRNA^KDR^-lipoplexes or siRNA^Luc^-lipoplexes, on consecutive days, starting immediately after portal hypertension induction. Studies and blood and organs collection were performed one day after the last injection.

For determination of blood chemistry parameters, blood samples were centrifuged at 3000 rpm for 10 min at 4 °C to obtain plasma. Alanine aminotransferase (ALT), aspartate aminotransferase (AST), albumin, creatinine and urea were subsequently evaluated by standard protocols using a Spinreact Spinlab 100 chemistry analyzer.

### Determination of the extent of portosystemic collateral formation

The extent of portosystemic collateral vessels was quantified using the coloured microsphere technique^7,34^. Under anesthesia with isofluorane (3.5% for induction; 1.5% for maintenance), a tracheostomy was performed and a polyethylene tubing (Clay-Adams Inc, New York, NY) was inserted into the trachea to ensure a patent airway. Then, an abdominal midline incision was performed. Approximately 150,000 yellow polystyrene latex microspheres (15-μm diameter, Dye-trak; Triton Technologies) were injected into the spleen of each animal. After waiting 5 min to allow a sufficient distribution of the microspheres through the whole body, mice were sacrificed and the lungs and liver were removed, cleaned and weighed. Microspheres were recovered from tissues by digestion and precipitation, following manufacture’s instructions. Blue microspheres (≈10,000) were used as control recovery in each tube. Dye concentration in microspheres was determined by spectrophotometry (670 nm for blue microspheres and 440 nm for yellow microspheres). Extent of collateralization was calculated as the percentage of microspheres in the lungs compared to lung and liver combined.

To estimate portosystemic collateral blood flow, the superior mesenteric artery was dissected free from connective tissue, and a nonconstrictive perivascular ultrasonic flowprobe (Transonic Systems, New York, NY) was placed around this vessel close to its aortic origin. The ultrasonic flowprobe was connected to a small animal T206 blood flowmeter (Transonic Systems) to measure blood flow in the superior mesenteric artery (SMABF, mL/min/10 g body weight) by the ultrasonic transit-time technique using ADI Chart software^9,10^. This method involves a perivascular flowprobe, which contains two ultrasonic transducers that emit a plane wave of ultrasound back and forth, alternatively intersecting the flowing blood in upstream and downstream directions. The flowprobe substracts the downstream from the upstream integrated transit times, and this difference is a measure of the volume of blood flow (mL/min). Rectal temperature was maintained at 37 ± 0.5 °C throughout the study. Portosystemic collateral blood flow (mL/min/10 g body weight) was estimated as SMABF × extent of portosystemic collaterals⁄100, as previously described^37^.

### Histological analyses and immunohistochemistry

Tissues were fixed in 10% buffered formalin solution and embedded in paraffin. Successive 2-μm sections were obtained and prepared for histological H&E staining, according to standard protocols. For immunostaining, after deparaffination and rehydration, sections were heated in a pressure cooker (in 10 mM citrate buffer, pH6.0, 5-min) for epitope retrieval, and treated with 3% hydrogen peroxide for 10-min at room temperature to inhibit endogenous peroxidase activity. Slides were blocked with 5% goat serum for 1 h, and then incubated with primary antibodies against KDR (1:50 dilution; sc-6251, Santa Cruz), Ki67 (1:25 dilution; ab16667, Abcam) or vWF (1:1000 dilution; A0082, DAKO) at 4 °C, overnight. Sections were then washed in TBST and incubated 30-min at room temperature with Dako Real EnVision Detection System (HRP mouse/rabbit secondary antibody). Antibody binding was revealed using hydrogen peroxide as substrate, and diaminobenzidine as chromogen. Hematoxylin was used as countestain. For negative control, primary antibody was omitted and sections were incubated with the corresponding secondary antibody and detection systems. Stained sections were visualized with a Zeiss microscope. Images from several regions of the tissue or vessel sections were then acquired using an AxioCam camera (Carl Zeiss Vision, Germany). Analysis of digitalized images was performed with computerized imaging system (AxioVision and Image J).

### Immunofluorescence and confocal laser microscopy

For *in vivo* uptake studies of fluorescently labeled siRNAs (Cy3-siRNAs), paraffin embedded 2-μm sections were deparaffined, rehydrated and stained with DAPI (H-1200 Vector Burlingame, CA). Then, sections were directly examined by epifluorescence with a confocal laser-scanning microscope. For coimmunostainings, paraffin sections were deparaffined, rehydrated, blocked with 5% goat serum and then incubated overnight at 4 °C with primary antibody against CD31 (1:50 dilution; sc-1506R; Santa Cruz). For fluorescence visualization of immunolabeling, a fluorescently labeled secondary (Alexa Fluor-647) was used (A31573; Invitrogen, Carlsbad, CA). Nuclei were counterstained with DAPI. Photomicrographs were taken with a confocal laser-scanning microscope, using QImaging digital camera and Imaging ProPlus software. All images were captured using similar confocal microscopy settings.

### Quantification of tissue vascularization

To quantify vascularization on tissue sections, blood vessels were first detected by vWF immunostaining. Digital images of different microscopic fields of each mesenteric tissue were then acquired using a Zeiss microscope and an AxioCam colour digital camera (Carl Zeiss Vision). Zeiss Axio Vision image analysis system (Zeiss) and IPLab software (BioVision Technologies) were used for computerized quantification of immunostained vascular structures. Total number of vWF-positive blood microvessels was counted accross entire mesenteric sections and divided by the section area to give total mean blood microvessel density for each group. Results were expressed as number of vessels per square millimeter. Sections were examined independently by 2 blinding investigators, experts in the field, who were unaware of the samples’ profiles.

### Determination of vascular endothelial cell proliferation

To identify proliferating endothelial cells in mesenteric vessels, we used monoclonal antibodies to the nuclear non-histone antigen Ki67. Total number of Ki67-positive endothelial cells per blood vessel was counted in high power fields (original magnification × 200) and expressed as percentage of the total number of endothelial cells in that field. Mitotically quiescent endothelial cells were easily recognized by the lack of Ki67 immunoreactivity, the counterstaining with hematoxylin (which stains the nuclei in blue) post-immunohistochemistry, and their location as a single cell layer lining the interior surface of blood vessels.

### Statistical analysis

Data are shown as mean ± SEM. Results that were normally distributed (*P* > 0.05 from Kolmogorov-Smirnov test) were compared with parametric statistical procedures (Student t test and ANOVA followed by Bonferroni’s test for multiple comparisons). Non-normally distributed results were compared with non-parametric tests (Kruskall-Wallis one-way ANOVA and Mann-Whitney-U test). Significance was accepted at *p* < 0.05.

All data generated or analysed during this study are included in this published article (and its Supplementary Information files).

## Results

### KDR-dependent endothelial tubulogenesis is blocked by siRNA^KDR^*in vitro*

**Figure 3:**
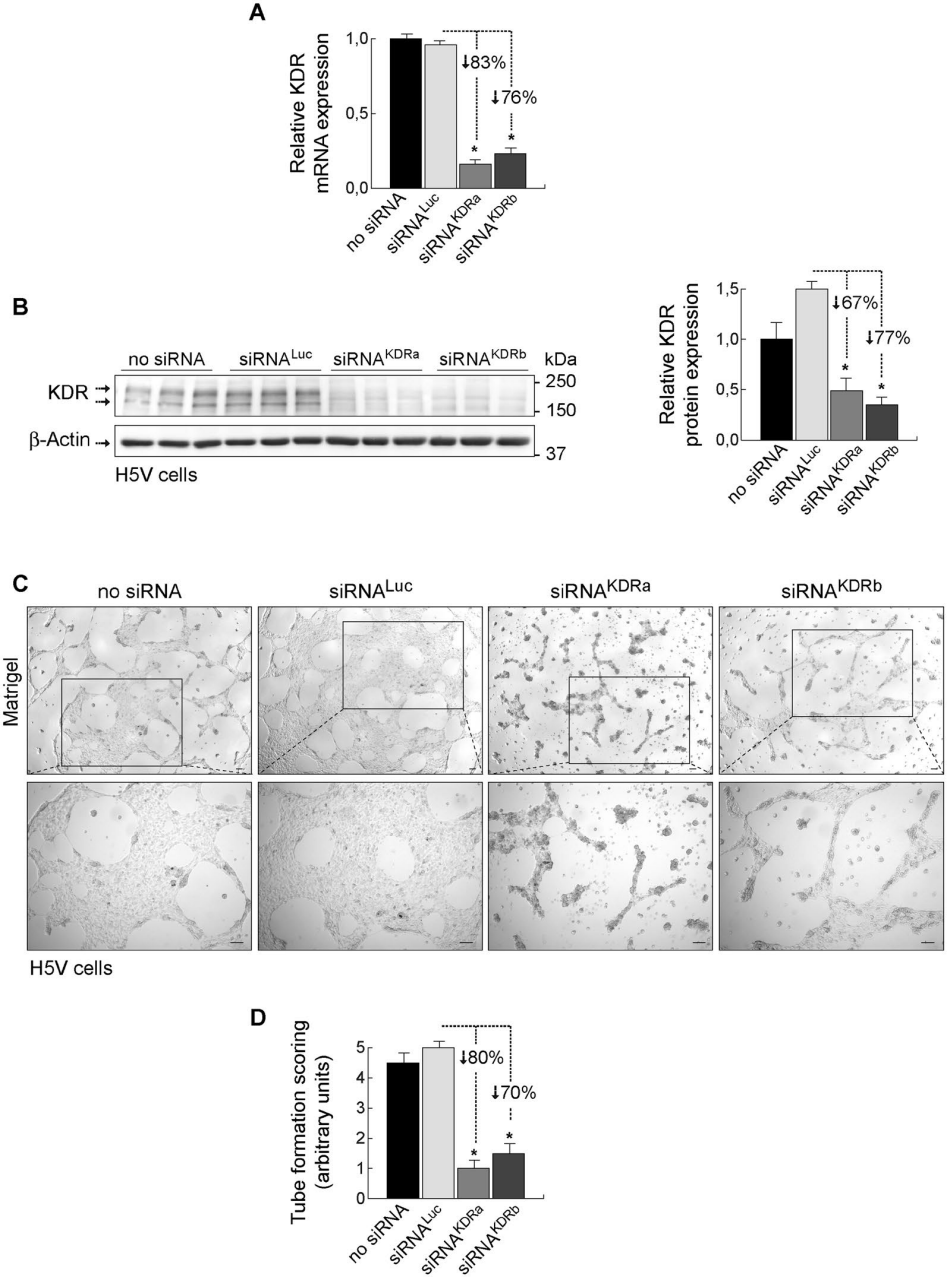
Loss-of-function analysis of KDR-specific siRNAs in murine endothelioma H5V cells. Murine endothelioma H5V cells were transfected with two different KDR targeting siRNA sequences (siRNA^KDRa^ and siRNA^KDRb^). Cells transfected with a luciferase-specific siRNA (siRNA^Luc^) or with empty liposome served as negative controls. **(A)** Relative KDR mRNA expression (mean ± SEM), normalized to endogenous control glyceraldehyde 3-phosphate dehydrogenase (GAPDH), determined by real-time RT-PCR in H5V cells. **(B)** Knockdown analysis for KDR protein expression determined by immunoblotting in H5V cells. Detection of β-actin served as control for equal protein loading. KDR immunoblots show the two typical monomeric bands at 230 kDa and 200 kDa, corresponding to partially and fully glycosylated KDR variants. Densitometric quantification of protein expression (mean ± SEM) is also shown. Whole blots are shown in Supplementary Fig. S2A. **(C)** *In vitro* angiogenesis assay in H5V cells seeded on Matrigel. Following transfection with siKDR, KDR knockdown H5V cells (i.e., H5V cells transfected with siRNA^KDR^) had diminished ability to form tubular structures compared with cells transfected with siRNA^Luc^ or empty liposome. Images at the bottom show high magnification of boxed area from top images. Scale bars: 100 μm. **(D)** Tube formation was evaluated and scored (mean ± SEM) as previously described^34^: (0) individual cells, well-separated; (1) cells begin to migrate and align themselves; (2) capillary tubes visible, no sprouting; (3) sprouting of new capillary tubes visible; (4) closed polygons begin to form; (5) complex mesh-like structures develop. *p < 0.01 *versus* siRNA^Luc^-transfected control cells.

To validate the efficacy of the designed siRNA^KDR^ sequences in downregulating KDR levels, we conducted initial functional *in vitro* experiments in primary human endothelial cells isolated from umbilical vein (described in Methods and Fig. 1), and also in the murine immortalized heart H5V endothelial cell line as an appropriate model of pathologic or activated endothelial cell^33–35^. H5Vs are transformed and tumorigenic endothelioma cells that differ considerably from their normal counterparts, being highly proliferative and constitutively expressing high levels of VEGF and KDR^33–35^. Mouse endothelioma H5V cells were transfected with two different siRNA sequences specifically targeting mouse KDR (siRNA^KDRa^ and siRNA^KDRb^). Cells transfected with a Luciferase-specific siRNA (siRNA^Luc^) and cells treated with empty liposome without siRNA (no-siRNA) served as controls. KDR mRNA expression was determined 48 h posttransfection by real-time PCR. We found that transfection with siRNA^KDR^ markedly decreased KDR mRNA by 83% (siRNA^KDRa^) and 76% (siRNA^KDRb^), compared with control siRNA^Luc^ (Fig. 3A). There was no difference in KDR mRNA expression between siRNA^Luc^-treated cells and cells treated with empty liposome. We corroborated specific decrease of KDR by immunoblotting. KDR protein levels were significantly reduced in H5V cells after treatment with siRNA^KDRa^ (67% decrease) and siRNA^KDRb^ (77% decrease), as compared with control siRNA^Luc^ (Fig. 3B **;** full blots are shown in Supplementary Fig. S2A). In agreement with data in the literature^38^, two monomeric bands were typically observed in KDR immunoblots at 230 kDa and 200 kDa, corresponding to partially and fully glycosylated KDR variants. β-Actin protein expression showed no difference between groups. These results revealed successful transfection of siRNA^KDR^ into activated H5V endothelial cells and specific reduction of KDR expression at the protein and mRNA levels.

We further studied the KDR loss-of-function phenotype by performing vasculogenic tube formation assays *in vitro* in H5V cells transfected with siRNA^KDR^, control siRNA^Luc^, or empty liposome. We found that knockdown of KDR following transfection with siRNA^KDR^ dramatically diminished the capability of H5V cells of forming network/tube-like structures on Matrigel *in vitro* (Fig. 3C and D), compared with controls. Thus, complex mesh-like structures with closed polygons developed only in H5V cells transfected with siRNA^Luc^ or empty liposome, but not in those transfected with siRNA^KDR^ (Fig. 3C and D). Tube formation was unaffected by the transfection of control siRNA^Luc^. Collectively, these data indicate that the reduction in KDR synthesis obtained by the siRNA^KDR^ transfection *in vitro* in activated endothelial cells resulted in the expected biological effects on KDR expression and endothelial tube formation.

### Specific uptake of siRNA-lipoplexes into vascular endothelium in portal hypertensive mice

**Figure 4:**
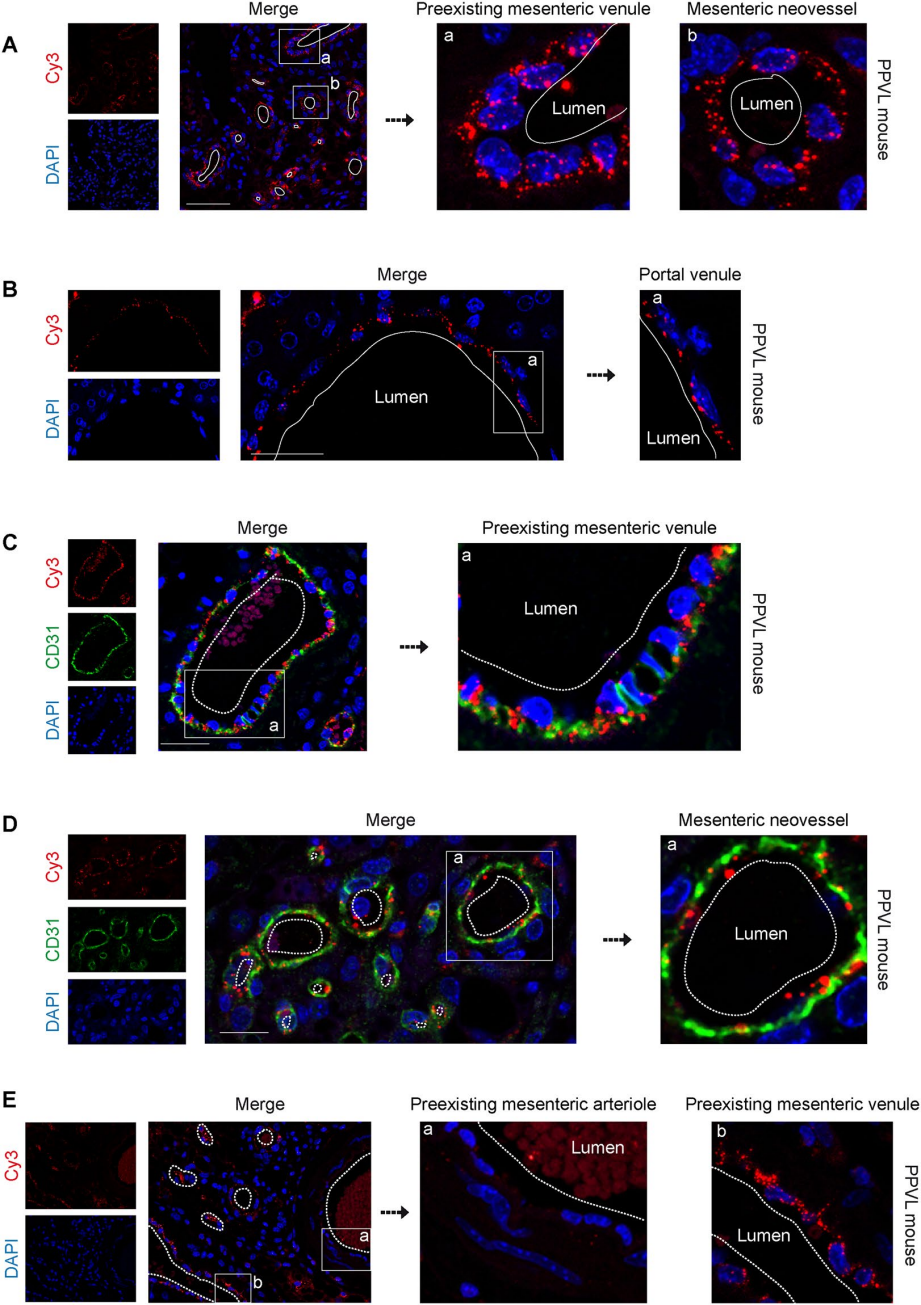
Intracellular localization of siRNA-Cy3-lipoplexes in the vascular endothelium of mesenteric microvessels and portal vein in portal hypertensive mice. The localization and cell-type specific uptake of lipoplexed siRNAs by the vascular endothelium *in vivo* was evaluated using fluorescently-labeled siRNA-Cy3-lipoplexes. These particles were intravenously injected in portal hypertensive mice, at day 5 after portal vein ligation (PPVL) and tissues were harvested 60 min later. Nuclei were stained with DAPI (blue). (**A**,**B**) High resolution confocal microscopy showing the intracellular localization of intravenously administered siRNA-Cy3-lipoplexes (Cy3 fluorescence; red) in vascular endothelial cells of preexisting microvenules and neovessels of the mesentery (**A**), as well as in the endothelium of intrahepatic portal venules (**B**). The Cy3-derived fluorescence was absent from non-vascular structures. Boxed areas are magnified to see that the distribution of the siRNA-Cy3-lipoplexes appears as punctuate fluorescent particles around the cell nuclei in the vascular endothelium. (**C**,**D**) The specific endothelial targeting shown in panels *A* and *B* was examined by double immunofluorescence of Cy3 and the endothelial cell marker CD31 and confocal microscopy. (**C**) Immunofluorescence staining with anti-CD31 antibodies showed that siRNA-Cy3-lipoplexes (red) were distributed along the CD31-positive endothelial cell lining (green) of the mesenteric preexisting microvenules, in the vicinity of the endothelial nucleus, further demonstrating the endothelial targeting. Close-up view from the indicated area of the merge is also shown. Note autofluorescent erythrocytes in the vessel lumen, and also the typical prominent nucleus of activated endothelial cells in mesenteric microvessels after portal hypertension induction. (**D**) The siRNA-Cy3-lipoplexes (red) also associated intimately with the CD31-labeled endothelial cells (green) of neovessels, suggesting again a strong internalization of these molecules to the microvascular endothelial cells in newly-formed mesenteric vessels. Nuclei were stained with DAPI (blue). Close-up view from the indicated area of the merge is also shown. (**E**) Mesenteric preexisting arteriole-venule pair showing poor or no fluorescent siRNA-Cy3-lipoplex labeling in the arteriole compared with microvenule and neovessels. Close-up views from the indicated area are also shown. Scale bars: 50 μm. See also Supplementary Fig. S3 (negative controls for immunofluorescence) and Supplementary Fig. S4 (H&E-stained mesenteric sections).

To address the therapeutic efficiency of siRNA^KDR^, we performed studies in a murine model of portal hypertension induced by partial portal vein ligation (PPVL). This is an ideal animal model to study pathological angiogenesis and portosystemic collateralization in the portal hypertensive syndrome, as it has been extensively demostrated^7–12^. For these *in vivo* experiments, we developed a novel siRNA delivery system based on AtuPLEX^TM^ technology (Silence Therapeutics, Berlin, Germany). siRNAs against KDR were chemically modified to strengthen their stability and ability to suppress the target gene effectively^27–32^. These therapeutic siRNAs were also encapsulated within liposomes to form siRNA lipoplex particles, which protect siRNAs from ribonucleases and shear degradation in the bloodstream, and facilitate their transfer across cellular membranes upon intravenous systemic administration. Due to specific lipid compositions, physico-chemical properties and cationic charge, AtuPLEX formulations confer targeting specifically to vascular endothelial cells, a prerequisite for mediating therapeutic KDR silencing^27–32,36^. Here we examined a new derivative of AtuPLEX, AtuPLEX2. To monitor the delivery of siRNA^KDR^-lipoplexes to the vascular endothelium *in vivo*, we used AtuPLEX2 lipoplexes formulated with cyanine dye (Cy3)-labeled nonsilencing siRNA. These siRNA-Cy3-lipoplexes were administered intravenously via tail-vein injection in mice, at day 5 after induction of portal hypertension. We have previously demonstrated the existence of a time-dependent increase in the amount of splanchnic blood vessels, with a clear upregulation of the endothelial cell marker CD31 at day 5 after portal hypertension induction in mice^7^. Tissues of interest (mesenteric microvasculature and portal vein draining from mesenteric veins, in which the proangiogenic VEGF-KDR signaling pathway plays a pivotal pathological role in portal hypertension^7–12^) were harvested 60 min later, and the red fluorescent Cy3-labeled siRNAs were visualized by confocal microscopy. We found considerable siRNA-related fluorescence in mesenteric vessels and intrahepatic portal vein (Fig. 4A and B; negative controls for immunofluorescence are shown in Supplementary Fig. S3). Upon close-upview, siRNA staining often appeared as punctuate structures around nuclei of vascular endothelial cells (Fig. 4A and B), this observation being indicative for intracellular uptake of siRNAs. To analyze in more detail the specific endothelial targeting of siRNA-Cy3-lipoplexes, we carried out double immunofluorescence of Cy3 and the endothelial cell marker CD31^39^. Confocal microscopy of mesenteric microvessels showed that the distribution of expression of siRNA-Cy3 and endogenous CD31 markedly overlapped in microvascular endothelium (Fig. 4C and D). Notably, siRNA-Cy3 accumulated mainly in the endothelium of newly-formed vessels and preexisting microvenules of the mesentery (Fig. 4A and C–E), while very little accumulation of these particles was observed in mesenteric microarterioles **(**Fig. 4E), even if these vessels are first exposed to the siRNA-liposomes before passing through capillaries and entering the venous circulation, considering the circulation route of liposomes post-tail vein administration. Furthermore, the Cy3-derived fluorescence was clearly absent from other mesenteric vascular cell types which are not endothelial cells (vascular smooth muscle cells and adventitial cells for example) and from non-vascular structures of the mesentery (such as adipocytes) (Fig. 4A–E), implying that the vasculature and, in particular the endothelial cells, were the preferred cellular target structures, minimizing exposure of other cells. See Supplementary Fig. S4 for illustration of the histology of mouse mesentery, highlighting the difference between preexisting vessels (preexisting microvenules and microarterioles), which readily distinguished from neovessels by their notoriously smaller caliber and thinner vascular wall. Furthermore, fluorescence was not detectable in mice not injected with siRNA-Cy3-lipoplexes, except for the autofluorescent erythrocytes in the vessel lumen, demonstrating that the light emissions observed in the mesenteries injected with labeled siRNA were not due to autofluorescence produced by the tissue (Supplementary Fig. S3). These findings confirm the capacity of the lipoplex formulation to efficiently deliver siRNA cargo *in vivo* to endothelial cells of mesenteric microvenules and portal vein.

### Effective silencing capability of siRNA^KDR^-lipoplexes in mice with portal hypertension

**Figure 5:**
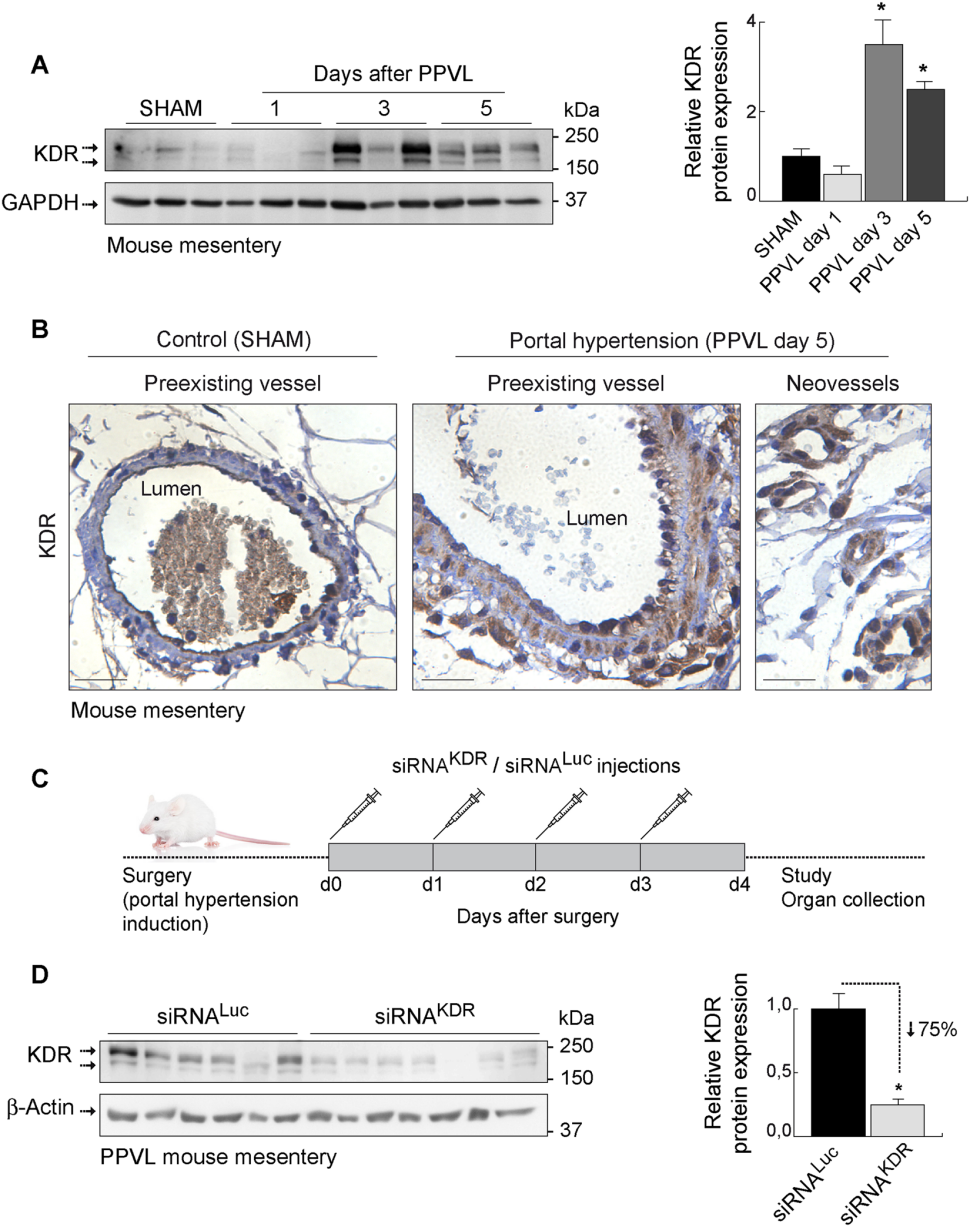
Knockdown of KDR overexpression after siRNA^KDR^-lipoplex treatment in mice. (**A**) KDR protein expression, determined by immunoblotting, in the mouse mesentery at different time points after portal hypertension induction by partial portal vein ligation (PPVL), and in sham-operated control mice (SHAM). GAPDH was used as loading control. Densitometric quantification of protein expression (mean ± SEM) is also shown. *P* value: *p < 0.05 *versus* SHAM mice. Whole blots are shown in Supplementary Fig. S2B. **(B)** Representative immunostainings showing the overexpression of KDR in preexisting microvenules and neovessels in the mesentery of portal hypertensive mice (PPVL day 5) versus SHAM control mice. Note that activated KDR-positive endothelial cells in portal hypertensive vessels are morphologically characterized by being rounded cells that protrude into the vessel lamina, in contrast to the exceedingly thin sheet of flattened resting endothelial cells observed under normal conditions. Scale bars: 25 μm. **(C)** Schematic representation of the schedule of siRNA-lipoplex treatment in mice. **(D)** Representative immunoblotting demonstrating effective reduction of KDR protein expression, by 75%, in the mesentery of siRNA^KDR^-lipoplex-treated portal hypertensive mice in comparison with siRNA^Luc^-lipoplex-treated portal hypertensive animals. β-Actin served as loading control. Densitometric quantification of protein expression (mean ± SEM) is also shown. *P* value: **p = 0.0014. Whole blots are shown in Supplementary Fig. S2C.

**Figure 6:**
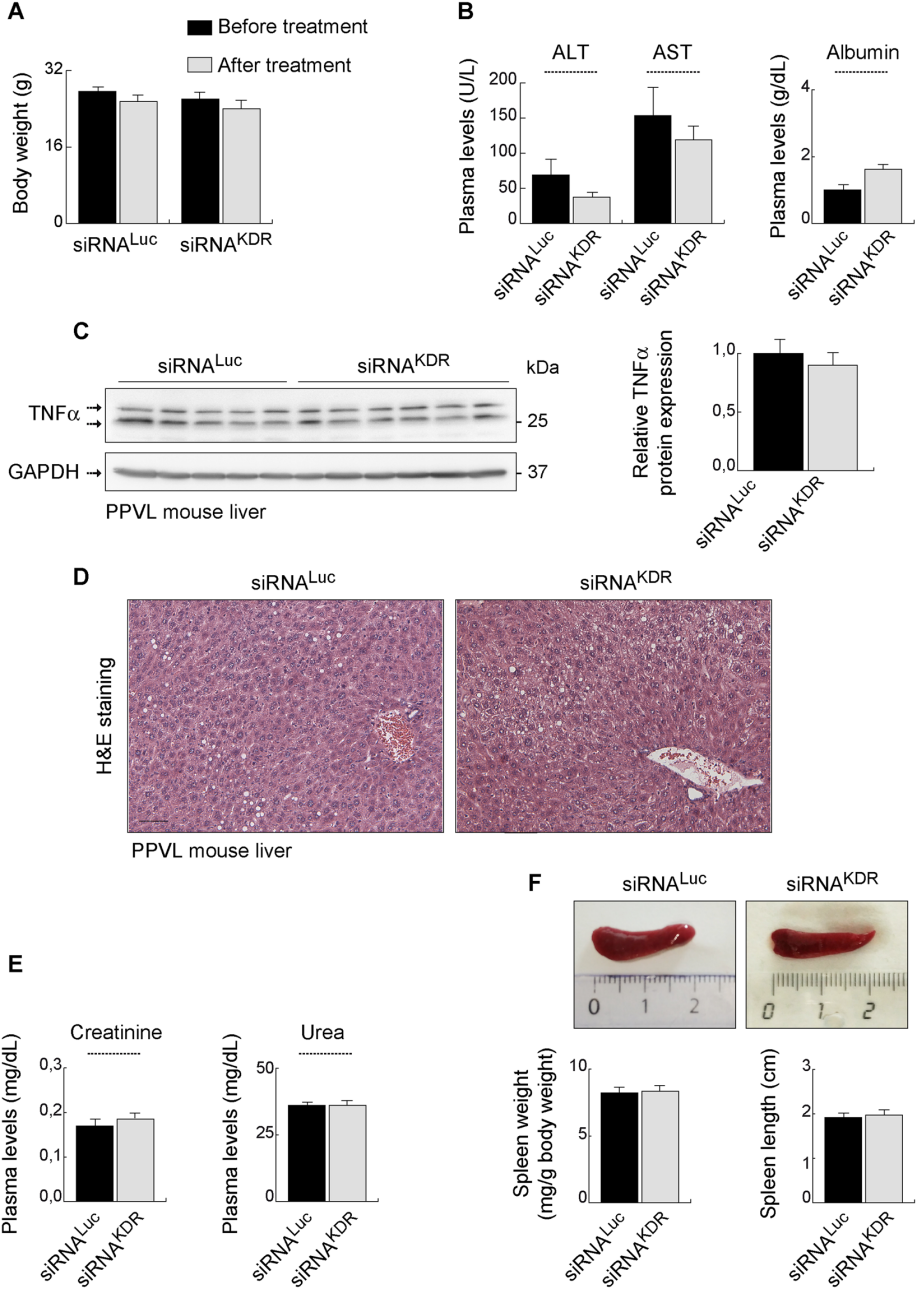
Lack of adverse effects after siRNA^KDR^-lipoplex treatment in mice. (**A**) Body weight (grams; mean ± SEM) in portal hypertensive mice, before and after treatment with siRNA^KDR^- and siRNA^Luc^-lipoplexes. **(B)** Plasma levels of alanine aminotransferase (ALT, U/L), aspartate aminotransferase (AST, U/L) and albumin (g/dL) in portal hypertensive mice treated with siRNA^KDR^- or siRNA^Luc^ lipoplexes. **(C)** Protein expression of the proinflammatory cytokine tumor necrosis factor-α (TNFα, determined by immunoblotting, in the liver of portal hypertensive mice treated with siRNA^KDR^- or siRNA^Luc^ lipoplexes. Densitometric quantification of protein expression (mean ± SEM) is also shown. Whole blots are shown in Supplementary Fig. S2D. **(D)** H&E staining in liver sections from portal hypertensive mice treated with siRNA^KDR^- or siRNA^Luc^ lipoplexes. **(E)** Plasma levels of creatinine (mg/dL) and urea (mg/dL) in portal hypertensive mice treated with siRNA^KDR^- or siRNA^Luc^ lipoplexes. **(F)** Spleen weight (mg/g body weight) and spleen length (cm) in portal hypertensive mice treated with siRNA^KDR^- or siRNA^Luc^ lipoplexes. Macroscopic photographs of representative spleens are also shown. All results are presented as mean ± SEM.

Having demonstrated the uptake of siRNAs by the specific cell type of interest, we further determined their ability to specifically knockdown the endogenous (over)expression of KDR, which is a hallmark of portal hypertension that contributes to disease progression and aggravation^6–12^. First, we confirmed that the expression of KDR was very low under baseline physiologic conditions in sham-operated control mice, but was strongly upregulated in mesenteric microvenules and neovessels upon portal hypertension induction (*p* < 0.05), as indicated by immunoblotting and immunohistochemistry (Fig. 5A and B; full blots are shown in Supplementary Fig. S2B). This KDR overexpression was efficiently and significantly reduced (by 75%, *p* = 0.0014) after systemic treatment with siRNA^KDR^-lipoplexes (four tail-vein injections on consecutive days, starting immediately after portal hypertension induction) (Fig. 5C), compared with mice treated with unrelated Luciferase siRNA lipoplexes (siRNA^Luc^-lipoplexes) (Fig. 5D **;** full blots are shown in Supplementary Fig. S2C). Notably, siRNA^KDR^-lipoplex treatment did not totally abolish expression of the KDR protein in mice with the current dosing regimen (Fig. 5D). Basically, KDR expression was knocked down but not knocked out, which is preferable to preserve basal levels of KDR to maintain vascular homeostasis of healthy vessels^16–20^. Accordingly, and under the conditions and dosage employed here, no gross side effects, for example no diarrhea, weakness or morbidity, were observed during the experiments. There were also no behavioral changes, such as eating and drinking habits and mobility in animals treated with liposomal siRNA preparations, both those that are nonsilencing and those targeting KDR. We identified comparable body weight loss in both groups (7.9% in the siRNA^KDR^-lipoplex group and 7.5% in the siRNA^Luc^-lipoplex group) (Fig. 6A). Note however that treatment runs in parallel with recovery from surgery, so this body weight loss can be due to the surgery and not to the siRNA lipoplex treatment. In addition, we found neither signs of altered liver function [alanine aminotransferase (ALT, *p* = 17), aspartate aminotransferase (AST, *p* = 0.37), and albumin (*p* = 0.24) plasma levels] nor evidence of inflammation or histologic toxicities in the liver [protein expression of the proinflammatory cytokine tumor necrosis factor-α (TNFα; *p* = 0.4), and H&E staining] after treatment with siRNA^KDR^-lipoplexes or control siRNA^Luc^-lipoplexes (Fig. 6B–D **;** full blots are shown in Supplementary Fig. S2D). There were also no signs of altered renal function [plasma levels of creatinine (*p* = 0.45), and urea (*p* = 1)] (Fig. 6E). The size of the spleen was also not affected by the treatments (Fig. 6F), which may be indicative of a lack of general inflammatory response^40^. Furthermore, the expressions of the proteins β-actin and GAPDH, used as loading controls, were unaffected in response to siRNA^KDR^-lipoplex treatment or in response to siRNA^Luc^-lipoplex treatment (Figs 5D and 6C), implying that there was no global cell loss or protein synthesis downregulation after siRNA administration. These findings together indicate that siRNA^KDR^-lipoplexes were not only intravenously delivered to the targeted vascular endothelial cells, but also reached sufficient therapeutic concentration to produce specific KDR knockdown, without adverse effects, in portal hypertensive mice. Of note, we have invested considerable effort in optimizing the RNA interference technology for functional studies and *in vivo* testing. Thus, all siRNAs used in this study were rigorously and thoroughly examined for potential off-target effects by bioinformatic analyses. Only sequences with 100% sequence identity to the target were selected; sequences with sequence similarity to other targets (with more than 16 out of 19 base pairs matches) were excluded; and all siRNAs were excluded when seed region matched to miRNA seed sequences (miRbase). Even more weight was put on the analysis of potential off-targets expressed in the vasculature. In addition, and as described in Methods, siRNAs were modified by alternating 2′O methyl modifications and 2′ fluoro modifications, which enhance siRNA stability and reduce Toll-like receptor activation and off-target activities. Furthermore, to exclude that target gene expression was modulated by the liposomal delivery system, control groups were included with liposomal formulations prepared with non-targeting siRNA payloads. Another concern that should be taken into consideration when using siRNAs for therapeutic or scientific purposes is the potential presence of some specific motifs in the siRNA sequence, such as UGGC- and other -AU-rich pentamers, including -AUUUG, GUUUU, AUUUU, CUUUU, UUUUU, GUUUG-, which may induce a toxic phenotype in cells, as described by Fedorov and colleagues^41^. One of the sequences used in our study (siRNA^KDR-hm2b^) contained an AUUUG motif. Of note, though, this motif was present only in the sense, passenger strand (GCAAGAGAAAUGAAUUUGU), but not in the antisense, guide strand (ACAAAUUCAUUUCUCUUGC) (Fig. 1C). This point is relevant because the potential toxicity of the AUUUG motif observed by Fedorov *et al*. occurred only when this was overrepresented in the RNA Induced Silencing Complex (RISC)-entering strand, the guide strand^41^. Strand loading of a siRNA duplex is determined by preferential loading of the antisense strand due to thermodynamic asymmetry. The sense strand (passenger strand) of a functional siRNA molecule has usually the more stable 5′-end than the antisense (guide strand). This is also the case with the siRNA^KDR-hm2b^: The antisense strand contains 3 GC base pairs in the first 5 nucleotides, compared to only one GC in the first 5 nucleotides of the antisense strand. The proposed “toxic motif” AUUUG of the siRNA^KDR-hm2b^ is however located in the sense strand of the siRNA duplex, which is not the preferred strand for RISC loading. To eliminate off-target effects, we have also adopted very stringent siRNA design filters and testing conditions, comparable or even more stringent than in Fedorov’s work^41^. Thus, while they tested their siRNAs in immortalized tumor cell lines, we tested our siRNA candidates in primary endothelial cell lines, which are considered to be a more relevant model for vascular biology and more sensitive to toxic stimuli. We did not observe cytotoxic effects at up to 20 nM siRNAs transfected. In Fedorov *et al*., toxic effects were observed at a 10 nM concentration (lipofectamine transfections)^41^. In addition, we used two different KDR siRNA sequences for *in vitro* phenotypic characterization. Even though the sequences were different, the same phenotype was observed in the Matrigel assay. Therefore, it is very unlikely that the *in vitro* and *in vivo* effects observed in our present study were due to the potential toxic sequence motifs.

### siRNA^KDR^-lipoplex therapy ameliorates portosystemic collateral growth and pathological neovascularity in portal hypertensive mice

**Figure 7:**
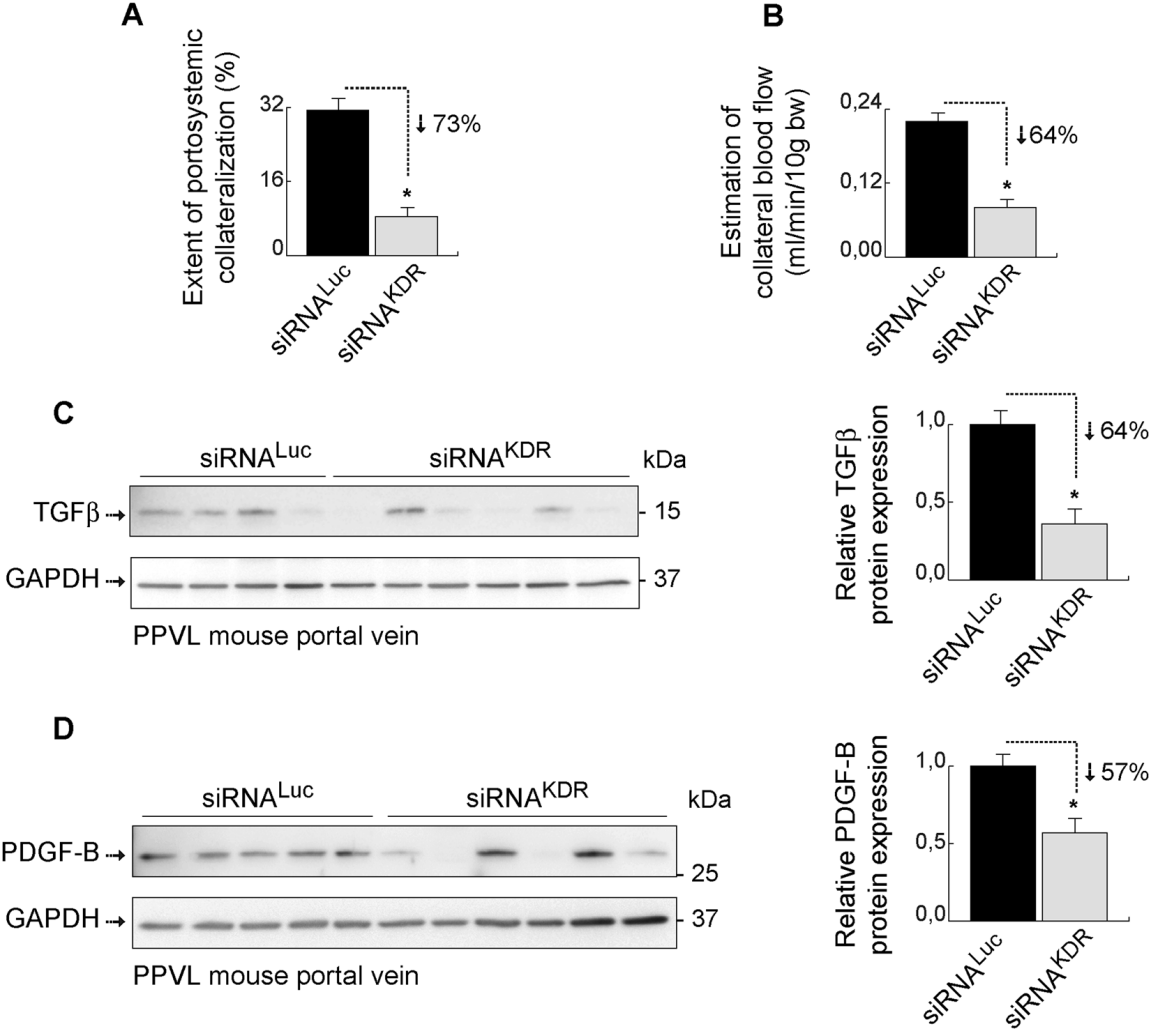
Therapy with siRNA^KDR^-lipoplexes attenuates portosystemic collateralization. Portal hypertensive mice were treated with four consecutive intravenous injections of siRNA^KDR^-lipoplexes or control siRNA^Luc^-lipoplexes after partial portal vein ligation (PPVL). **(A)** Extent of portosystemic collateralization (%; mean ± SEM) determined by the coloured microsphere technique. *P* value: *p = 0.0002. **(B)** The blood flow through portosystemic collaterals (mL/min/10 g body weight; mean ± SEM) was estimated from the equation collateral blood flow = superior mesenteric artery blood flow × shunting/100. *P* value: *p = 0.0049. Treatment with siRNA^KDR^-lipoplexes markedly and significantly decreased both the formation of portosystemic collateral vessels, by 73%, and the collateral blood flow, by 64%, in portal hypertensive mice. **(C)** Representative immunoblotting showing that siRNA^KDR^-lipoplex treatment reduced in 5 out of 6 mice the protein expression of the vascular remodeling marker TGFβ (average decrease of 64%; *P* value: *p = 0.041) in the portal vein, compared with siRNA^Luc^-lipoplex-treated portal hypertensive mice. GAPDH served as loading control. Densitometric quantification of protein expression (mean ± SEM) is also shown. Whole blots are shown in Supplementary Fig. S5A. **(D)** Representative immunoblotting showing that siRNA^KDR^-lipoplex treatment reduced in 4 out of 6 mice the protein expression of the vascular remodeling marker PDGF-B (average decrease of 57%; *P* value: *p = 0.029) in the portal vein, compared with siRNA^Luc^-lipoplex-treated portal hypertensive mice. GAPDH served as loading control. Densitometric quantification of protein expression (mean ± SEM) is also shown. Whole blots are shown in Supplementary Fig. S5B.

We next proceeded to specifically analyze the effects and therapeutic potential of siRNA^KDR^-lipoplexes on the portosystemic collateral circulation. To this end, mice were subjected to PPVL to induce portal hypertension, and then treated as previously indicated with four consecutive tail-vein injections of siRNA^KDR^-lipoplexes or control siRNA^Luc^-lipoplexes. The extent of portosystemic collateralization was determined by the tracer microsphere technique, as described in the Methods section. This well-established method uses the injection of microspheres into the spleen to measure portosystemic collaterals. When no collaterals are detectable, all microspheres go directly to the liver through the portal vein and remain retained in the liver. However, when portosystemic collateral vessels have developed, microspheres bypass the liver and circulate through the portosystemic collaterals to the lungs, where they remain retained. We found that therapy with siRNA^KDR^-lipoplexes, initiated early in the course of portal hypertension progression, drastically reduced by 73% the portosystemic collateralization (*p* = 0.0002), compared with siRNA^Luc^-lipoplex treatment (Fig. 7A). Of note, 80% of the animals in the siRNA^KDR^-lipoplex group had an almost negligible (<10%) portosystemic collateralization. In comparison, only 6% of the mice in the siRNA^Luc^-lipoplex group had collateralization of <10%. This effect was paralleled by a marked and significant decrease (by 64%; *p* = 0.0049) in collateral blood flow (Fig. 7B), which can additionally reduce the functional capacity of collaterals by modulating vascular remodeling^5^. It must be noted that the endothelial cell surface KDR receptor is able to perceive changes in blood flow in a ligand-independent manner, acting as a mechanoreceptor that triggers vessel remodeling^42–45^. Such remodeling, favoured by increased collateral blood flow, is an important mechanism mediating the enlargement and maintenance of collateral vessels in portal hypertension^5,23^. In this respect, we found that siRNA^KDR^-lipoplex treatment reduced the expression of the recognized vascular remodeling markers^46–48^, transforming growth factor-β (TGFβ, 64% decrease, *p* = 0.041) (Fig. 7C **;** full blots are shown in Supplementary Fig. S5A) and platelet-derived growth factor-B (PDGF-B; 57% decrease, *p* = 0.029) (Fig. 7D **;** full blots are shown in Supplementary Fig. S5B), in the portal vein of portal hypertensive mice.

**Figure 8:**
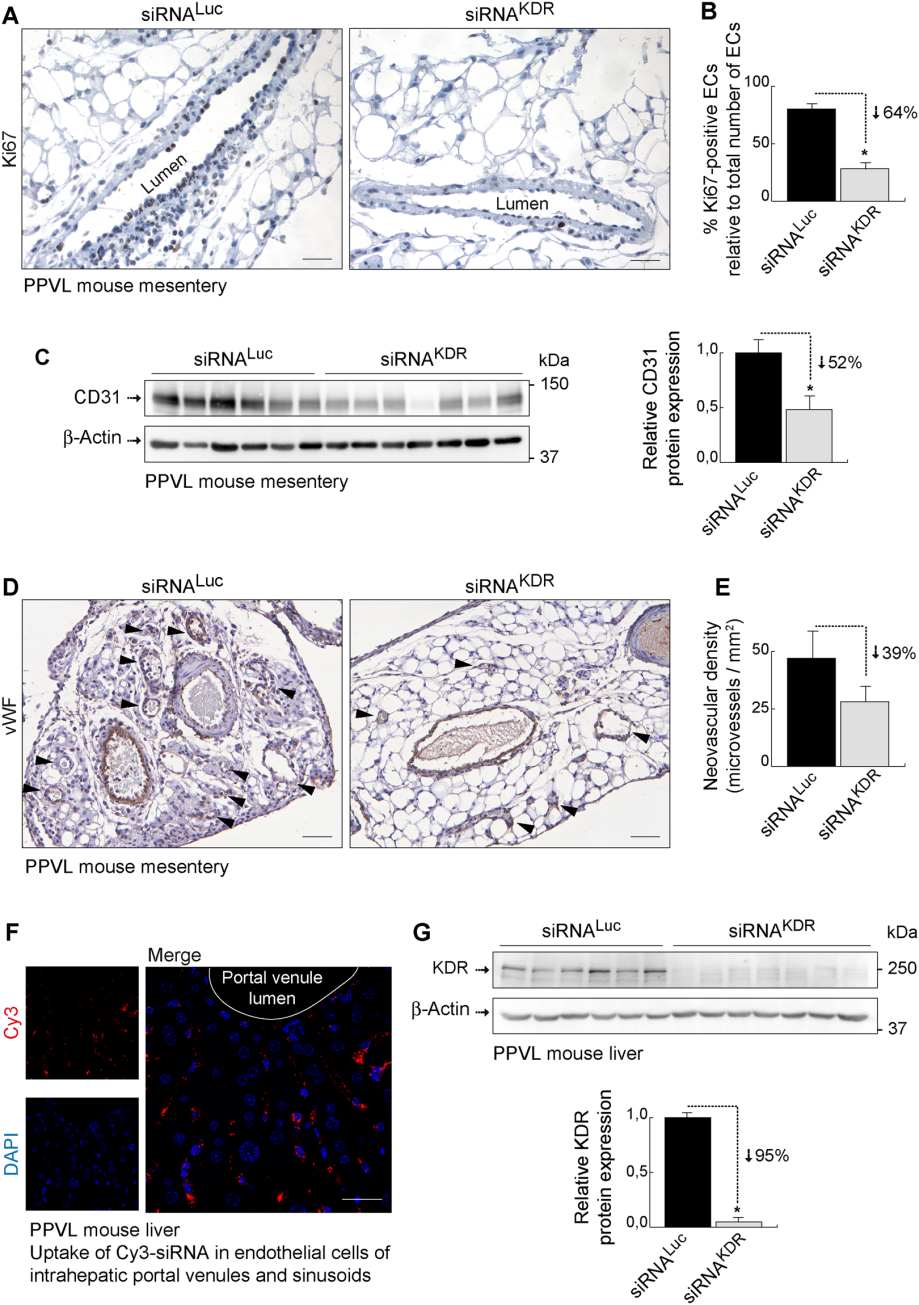
siKDR therapy limits pathological angiogenesis. (**A**) Representative Ki67 (cell proliferation marker) immunostaining in the mesentery of siRNA^KDR^-lipoplex-treated and siRNA^Luc^-lipoplex-treated portal hypertensive mice. Preexisting mesenteric microvenules with actively dividing endothelial cells were observed in siRNA^Luc^-lipoplex-treated portal hypertensive mice. Treatment with siRNA^KDR^-lipoplexes markedly reduced endothelial cell proliferation. Scale bars: 25 μm. **(B)** Graph showing the percentage of total vascular endothelial cells that are positive for the cell proliferation marker Ki67 in each experimental group (%; mean ± SEM). Mitotically quiescent endothelial cells were easily recognized by the lack of Ki67 immunoreactivity, the counterstaining with hematoxylin (which stains the cell nuclei in blue) post-immunohistochemistry, and their location as a single cell layer lining the interior surface of the blood vessels. *P* value: *p < 0.05. **(C)** Representative immunoblotting showing decreased protein expression of the endothelial cell marker and angiogenesis index CD31 in the mesentery from siRNA^KDR^-lipoplex-treated portal hypertensive mice, in comparison with siRNA^Luc^-lipoplex-treated portal hypertensive animals. β-Actin served as loading control. Densitometric quantification of protein expression (mean ± SEM) is also shown. *P* value: *p = 0.0022. Whole blots are shown in Supplementary Fig. S5C. **(D)** Representative von Willebrand factor (vWF; endothelial cell marker) immunostaining in the mesentery of siRNA^KDR^-lipoplex-treated and siRNA^Luc^-lipoplex-treated portal hypertensive mice. Arrowheads indicate neovessels, which readily distinguished from preexisting vessels by their notoriously smaller caliber and thinner vascular wall. Scale bars: 50 μm. **(E)** Quantification of vascular density (vWF-positive vessels/mm^2^; mean ± SEM) of neovessels in mouse mesentery. **(F)** High resolution confocal microscopy showing the intracellular localization of fluorescently-labeled siRNA-Cy3-lipoplexes (red) in the endothelium of intrahepatic sinusoids and portal venules in portal hypertensive mice. Nuclei were stained with DAPI (blue). Scale bars: 50 μm. **(G)** Representative immunoblotting demonstrating effective reduction of KDR protein expression, by 95%, in the liver of siRNA^KDR^-lipoplex-treated portal hypertensive mice in comparison with siRNA^Luc^-lipoplex-treated portal hypertensive animals. β-Actin served as loading control. Densitometric quantification of protein expression (mean ± SEM) is also shown. *P* value: *p < 0.0001. Whole blots are shown in Supplementary Fig. S5D.

Consistent with these findings, endothelial-specific KDR knockdown in portal hypertensive mice impaired the angiogenic potential of endothelial cells and reduced pathological neovascularization in the mesenteric vascular bed as well. Thus, treatment with siRNA^KDR^-lipoplexes caused a remarkable 64% diminution (*p* < 0.001) in endothelial cell proliferation (Fig. 8A and B), which precedes the development of new microvessels, as indicated by quantitative analysis of immunostaining with Ki67, a nuclear protein expressed only in proliferating cells, but not in dormant cells^49^. This effect was paralleled by a significant 52% decrease (*p* = 0.0022) in the protein expression of CD31 (Fig. 8C; full blots are shown in Supplementary Fig. S5C), measured by immunoblotting as a marker for endothelial cells^39^, and a tendency to attenuate (by 39%) mesenteric microvascular density (Fig. 8D and E), as evaluated by histomorphometric analysis of mesenteric sections immunostained for the vessel specific marker, von Willebrand factor (vWF)^43^. However, although many siRNA^KDR^-lipoplex-treated mice had lower density of microvessels than animals receiving siRNA^Luc^-lipoplex, averaged microvascular density was not significantly different between both treatment groups (*p* = 0.3), within the experimental parameters of the present study. A possible explanation comes from our recent findings^50^. According to them, the abnormal neovascularization of the mesentery in portal hypertension arises through combination and proper coordination of both angiogenesis (endothelial cell-dependent and therefore targeted by siRNA^KDR^-lipoplexes) and vasculogenesis (dependent on vascular wall-resident stem progenitor cells and therefore not directly targeted by the therapy used in this study)^50^.

Treatment with siRNA^KDR^-lipoplexes did not significantly modify spleen size (spleen weight and length per body weight ratio) (Fig. 6F), which is an indirect surrogate marker of portal pressure^40^, indicating that inhibition of collateralization by siRNA^KDR^-lipoplex administration occurred independently of the increased portal venous pressure, in agreement with our previous findings^7,8^.

In this study, we focused our attention on the effects of a siRNA^KDR^-lipoplex therapy on the portosystemic collateral circulation in portal hypertension, and selected the partial portal vein ligated model as an ideal animal model to study this specific disturbance. This is a model of prehepatic portal hypertension that develops a large percentage of portosystemic shunting, but not the typical abnormalities of the intrahepatic microcirculation in cirrhosis. But here we wanted to determine at least the potentiality of the siRNA^KDR^-lipoplexes to target the liver. Indeed, we found that siRNA-lipoplexes were avidly and effectively uptaken by intrahepatic endothelial cells (both endothelial cells of portal venules in portal tracts and sinusoidal endothelial cells) (Fig. 8F), and were able to silence KDR expression (*p* < 0.001) (Fig. 8G; full blots are shown in Supplementary Fig. S5D), without significantly affecting liver function, as described above (Fig. 6B–D).

## Discussion

In the present study, we have developed an innovative siRNA delivery system based on clinical stage components (i.e., siRNA^KDR^-lipoplexes), capable to efficiently and specifically target KDR in vascular endothelial cells *in vivo* by systemic intravenous administration. Our results demonstrate that therapy with siRNA^KDR^-lipoplexes is a highly promising approach to control and minimize the formation of portosystemic collateral vessels in portal hypertension. Thus, portosystemic circulation was highly vulnerable and susceptible to a treatment with siRNA^KDR^-lipoplexes, initiated early in the course of the disease^7^, leading to a dramatic prevention of collateral extent and impairment of the angiogenic potential of endothelial cells in a murine model of portal hypertension. We also found that the mechanisms underlying the effects of KDR knockdown include reduced endothelial cell proliferation and decreased angiogenesis and vascular remodeling.

These findings are of particular significance since, although much has been learned about the pathophysiology of portosystemic collateral growth and the role that angiogenesis and KDR plays on it^7–10^, this scientific knowledge has not yet been translated into clinical application. As a consequence, the development of portosystemic collaterals and gastroesophageal varices still remains one of the most clinically devastating consequences of portal hypertension and chronic liver disease^1–5^. Importantly, targeting KDR with siRNA^KDR^-lipoplexes could be a promising and plausible therapeutic modality for attenuating the formation of portosystemic collateral vessels in a clinical setting. Of note, this therapeutic intervention may potentially prevent the formation of large varices from small varices. This is important because new collaterals and varices develop each year during the evolution of chronic liver disease, and, currently, no treatment can prevent this development. Of interest, a related type of formulation has been shown to be viable and well tolerated for systemic administration of siRNAs and it is currently being evaluated in clinical trials for treatment of patients with advanced solid tumors, further supporting the translational relevance and therapeutic potential of this approach for portal hypertension and chronic liver disease in a clinical context.

The aim of targeting KDR in vascular endothelial cells in this study was based on three major observations: i) the well-established central role of endothelial KDR in pathological angiogenesis^16–20^, ii) prior work linking KDR to portosystemic collateralization in portal hypertension^7–10^, and iii) the ease by which endothelial cells can be targeted by siRNAs due to their unique position in the vessel and direct accessibility for systemically administered siRNA-loaded particles. In fact, our siRNA^KDR^-lipoplexes were designed to allow systemic delivery specifically to vascular endothelial cells^27–32^. This is favorable by reducing both the amounts of siRNA needed for therapeutic benefit as well as the likelihood of off-target effects caused by KDR knockdown in other cell types. Notably, we achieved an efficient functional intracellular siRNA^KDR^ delivery after intravenous administration in portal hypertensive mice, being the siRNA-lipoplexes avidly taken up by the angiogenic microvasculature. Interestingly, the delivery efficiency was higher in the activated endothelium of mesenteric microvenules of portal hypertensive mice than in microarterioles. This is relevant because sprouting angiogenesis typically originates from the venous side of the vascular bed^16,17^, and our previous studies demonstrate that the majority of changes associated with mesenteric neovascularization in portal hypertension affect the microvenules and, to a lesser degree, the microarterioles^34,48^. It must be noted that endothelial cells in portal hypertensive mesenteric microvenules have an activated phenotype characterized by overexpression of KDR, expression of cell proliferation markers (Ki67) and a rounded morphology with prominent nucleus (Figs 4 and 5B)^34,49^. In addition, structural and functional differences among arterioles and venules are reflected by unique transcriptional signatures within microvascular endothelial cells lining these vessels^51^. It is therefore likely that this portal hypertension-activated angiogenic microvenular endothelium was especially predisposed to uptake siRNA-lipoplexes, which is essential in order to achieve therapeutic effects.

Consequently, our delivery strategy allowed us to achieve subtantial reduction of the pathologically relevant KDR overexpression in portal hypertensive mice, of a sufficient magnitude to induce therapeutic effects, but not a complete dissapearance of KDR indicating that some functional RNA or protein remains and is translated at lower levels. This is beneficial for molecular targets such as KDR, which are crucial for vascular homeostasis^16–20^. Furthermore, the use of siRNA^KDR^ would also allow selective KDR inhibition without affecting even closely related proteins compared with the relative promiscuity of small-molecule inhibitors and multikinase inhibitors^8–10^. Worthy of note is that the RNA interference pathway, used by siRNA molecules to selectively limit the expression of targeted mRNAs, naturally occurs in the organism^24–26^.

Another interesting aspect is that KDR is not only a master controller of angiogenic events such as endothelial cell proliferation, sprouting and branching^16–20^, but is also an active player in modulation of vasomotor collateral dynamics and vascular remodeling^42–45^, which are additional important driving forces for collateral growth in portal hypertension^5,42,43^. In fact, KDR can promote remodeling independently of its VEGF-binding capability, by acting directly as a mechanoreceptor sensitive to changes in blood flow and shear stress^42–45^. Accordingly, targeting KDR would offer the added value of suppressing portosystemic collateralization on several levels, such as angiogenesis and flow-induced collateral remodeling, as suggested by the striking magnitude of reduction in portosystemic collateralization and collateral blood flow, as well as the downregulation of the expression of vascular remodeling markers produced by the siRNA^KDR^-lipoplex therapy in portal hypertensive mice.

In summary, our preclinical studies with siRNA^KDR^-lipoplex in a mouse model demonstrate a therapeutic benefit in preventing the development of portosystemic collateral vessels in portal hypertension. Overall, we believe that our study represents an important new step towards the application of siRNAs as therapeutic agents in portal hypertension and chronic liver disease. Given the emerging roles of angiogenesis in a number of human pathologies, including inflammation, obesity and tumor growth, siRNA^KDR^-lipoplexes may provide a novel strategy to treat a wide spectrum of diseases.

## Electronic supplementary material

Supplementary Information
