# Supplementary material for: Therapeutic siRNA targeting endothelial KDR decreases portosystemic collateralization in portal hypertension
Source: Sci Rep. 2017 Nov 1;7:14791. doi: 10.1038/s41598-017-14818-7 (PMC5665956; doi:10.1038/s41598-017-14818-7)
Supplement: Supplementary file 1 — Supplementary Information [file 41598_2017_14818_MOESM1_ESM.pdf]

SUPPLEMENTARY INFORMATION

Therapeutic siRNA targeting endothelial KDR decreases portosystemic collateralization in portal hypertension

Javier Gallego, Ester Garcia-Pras, Marc Mejias, Nuria Pell , Ute Schaeper, Mercedes Fernandez

SUPPLEMENTARY FIGURE LEGENDS

Supplementary Fig.S1: Full blots corresponding to western blottings in Main Figure 1: **(A)** Full blots for KDR and its loading control p110α in HUVEC cells, corresponding to western blotting in Figure 1A. **(B)** Full blots for KDR and its loading control p110α in HUVEC cells, corresponding to western blotting in Figure 1B (top). **(C)** Full blots for KDR and its loading control p110α in HUVEC cells, corresponding to western blotting in Figure 1B (bottom).

Supplementary Fig.S2: Full blots corresponding to western blottings in Main Figures 3, 5 and 6: **(A)** Full blots for KDR and its loading control β-actin in H5V cells, corresponding to western blotting in Figure 3B. **(B)** Full blots for KDR and its loading control GAPDH in mouse mesentery, corresponding to western blotting in Figure 5A. **(C)** Full blots for KDR and its loading control β-actin in PPVL mouse mesentery, corresponding to western blotting in Figure 5D. **(D)** Full blots for TNFα and its loading control GAPDH in PPVL mouse liver, corresponding to western blotting in Figure 6C.

Supplementary Fig.S3: Negative controls for immunofluorescence: (A) Representative confocal microscopy micrographs of the mesentery from portal hypertensive mice (at day 5 after partial portal vein ligation, PPVL). These mice were not injected with siRNA-Cy3-lipoplexes, and were used as tissue autofluorescence negative controls to test whether the light emissions observed in the mesenteries injected with labeled siRNA were due to autofluorescence produced by the tissue. **(B)** In negative controls for double immunofluorescence

stainings, mice were not injected with siRNA-Cy3-lipoplexes and the anti-CD31 primary antibody was ommitted. In both cases, not detectable fluorescence was found in these tissues, except for the autofluorescent erythrocytes in the vessel lumen. Scale bars: 50 μm.

Supplementary Fig.S4: Histology of mouse mesentery: Representative micrograph of mesenteric sections stained with H&E from portal hypertensive mice, illustrating preexisting vessels (preexisting venule and preexisting arteriole) and also neovessels (arrowheads), which are evident already at day 3 after inducing portal hypertension by partial portal vein ligation (PPVL). Image also shows adipocytes, which are the largest component of loose connective tissue of the mesentery. Note that adipocytes appear empty as lipids are extracted during specimen preparation. Scale bar: 50 μm.

Supplementary Fig.S5: Whole blots corresponding to western blottings in Main Figures 7 and 8: **(A)** Full blots for TGFβ and its loading control GAPDH in PPVL mouse portal vein, corresponding to western blotting in Figure 7C. **(B)** Full blots for PDGF-B and its loading control GAPDH in PPVL mouse portal vein, corresponding to western blotting in Figure 7D. **(C)** Full blots for CD31 and its loading control β-actin in PPVL mouse mesentery, corresponding to western blotting in Figure 8C. **(D)** Full blots for KDR and its loading control β-actin in PPVL mouse liver, corresponding to western blotting in Figure 8G.

SUPPLEMENTARY METHODS

RNA isolation and real-time reverse transcriptase (RT)-PCR analysis. Total RNA was extracted and purified from mouse H5V cells using the RNAeasy Mini Kit (Quiagen), in accordance with the manufacturer's instructions. The amount of RNA was measured by NanoDrop ND-1000 spectrophotometer (NanoDrop Technologies, Wilmington, DE). Then, 1 μg of total RNA was reverse transcribed into complementary DNA (cDNA) using the QuantiTect Reverse Transcription kit (Qiagen, Hilden, Germany), which includes all the components required for effective elimination of genomic DNA contamination in RNA samples and for accurate real-time PCR. The synthesized cDNA was then amplified by real time PCR using KDR (Mm01222421_m1) and GAPDH (Mm99999915_g1) TaqMan Gene expression assays (Applied Biosystems, Carlsbab, CA), in an ABI PRISM 7900 Sequence Detection System (Applied Biosystems). Each sample was run in duplicate using nuclease-free water as control. Expression levels of KDR gene were normalized to endogenous control GAPDH, which was unaffected in the different treatment groups.

Immunoblotting. Mouse tissues were excised, immediately snap-frozen in liquid nitrogen and pulverized in a ceramic mortar surrounded by dry ice. Aliquots of the powder were then homogenized in ice-cold lysis buffer containing 20 mM Tris, pH 7.5, 150 mM NaCl, 10 μg/mL aprotinin, 10 μg/mL leupeptin, 10 μg/mL pepstatin, 40 μg/mL phenylmethylsulfonyl fluoride, 10 μM sodium pyrophosphate, 20 μM sodium fluoride, 1% Triton X-100, 1 mM sodium orthovanadate, and 0.1 μM okadaic acid. The resultant lysates were centrifuged, the supernatants were

collected, and total protein concentration was measured using the Bradford Protein assay (BioRad; Hercules, CA). Equal amounts of total protein (25 μg) were heated (5 min at 95ºC) in sample buffer containing sodium dodecyl sulfate (SDS) and β-mercaptoethanol, and separated by SDS-polyacrylamide gel electrophoresis (SDS-PAGE). After transfer onto polyvinylidene fluoride (PVDF) membranes, specific proteins were incubated, at 4°C overnight, with monoclonal primary antibodies against KDR (1:200 dilution; sc-6251, Santa Cruz Biotechnology, Santa Cruz, CA) and PDGF-B (1:500 dilution; sc-365805, Santa Cruz) and polyclonal primary antibodies against TNFα (1:1000 dilution; ab66579, Abcam), CD31 (1:200 dilution; sc-1506R, Santa Cruz) and TGFβ (1:500 dilution; ab66043, Abcam). Blots were subsequently washed and incubated with horseradish peroxidase-conjugated secondary antibodies (1:10,000 dilution): anti-rabbit IgG (ENZO, Farmingdale, NY) and anti-mouse IgG (Sigma, Saint Louis, MO). Immunoreactive bands were detected using an enhanced chemiluminescence detection system (Immobilion Western; Millipore, Billerica, MA). Loading accuracy was evaluated by Ponceau S staining and by membrane rehybridization with mouse monoclonal antibodies against β-actin (1:1,000 dilution; A2228, Sigma) or glyceraldehyde 3-phosphate dehydrogenase (GAPDH; 1:1,000 dilution; sc-32233, Santa Cruz). Quantification of protein signals was performed using computer-assisted densitometry. For immunoblotting in cells, cells were collected by trypsinization and lysed with the ice-cold lysis buffer described above. After a strong vortex, lysates were centrifuged (10 min, at 4ºC and maximum speed) and supernatants were collected, quantified, and desnaturalyzed. Equal amounts of proteins were immunoblotted as described above. Antibodies against human KDR used in HUVECs were from Cell Signaling (#2479; Danvers, MA).

A Full blots corresponding to Figure 1A

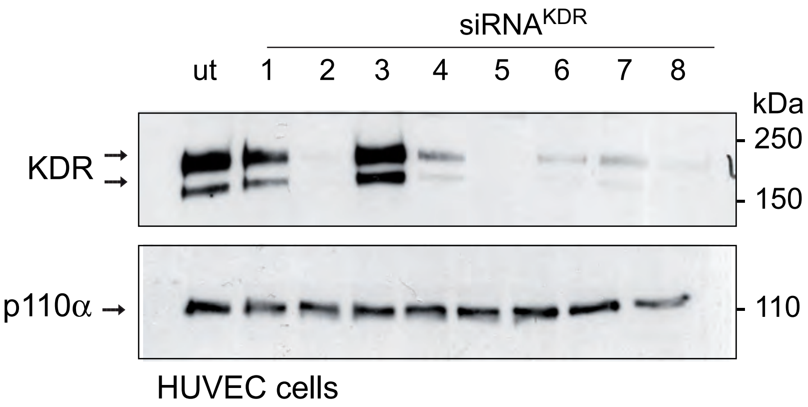

B Full blots corresponding to Figure 1B (top)

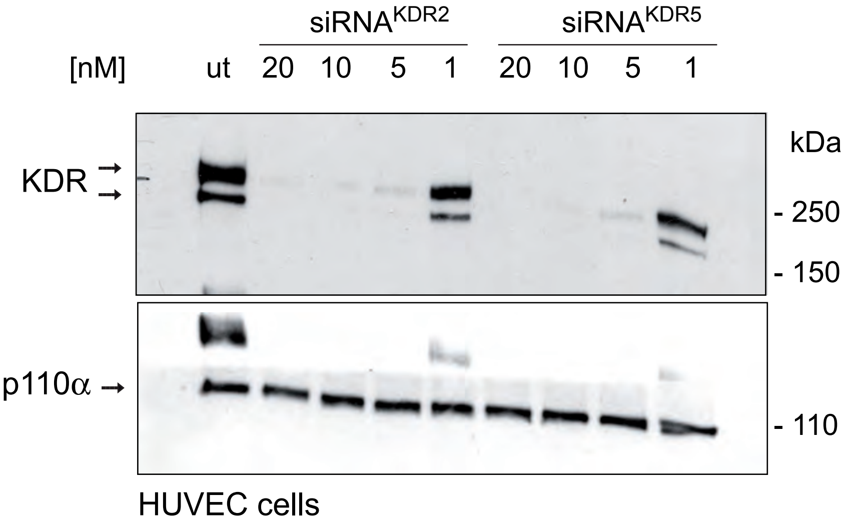

C Full blots corresponding to Figure 1B (bottom)

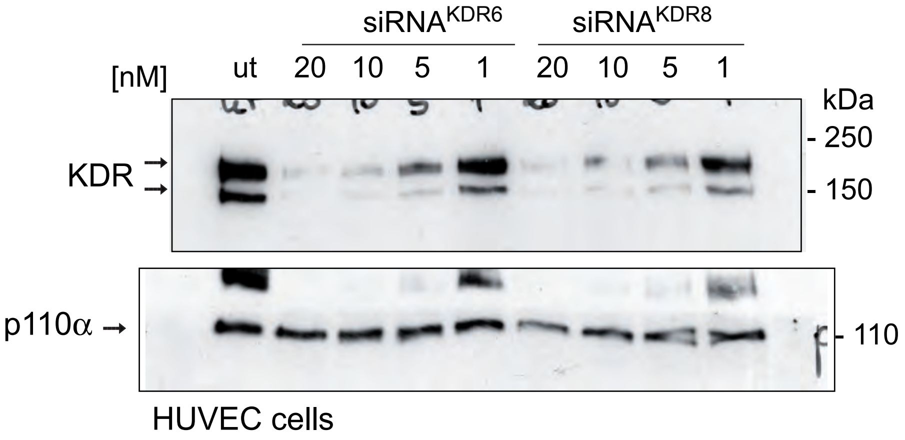

A Full blots corresponding to Figure 3B

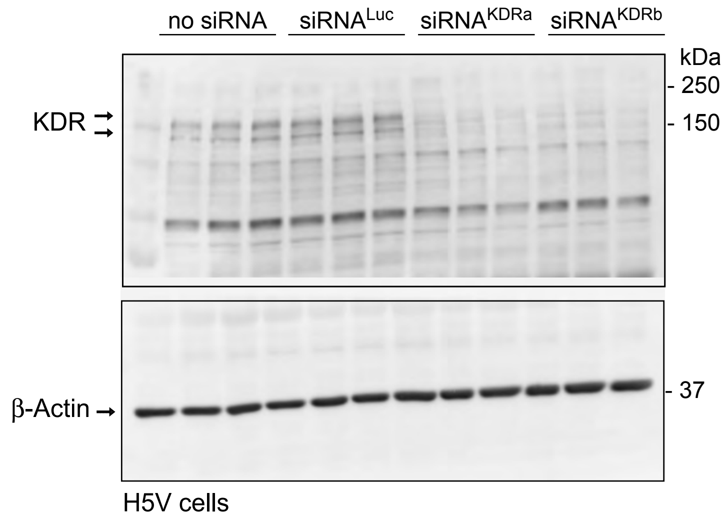

B Full blots corresponding to Figure 5A

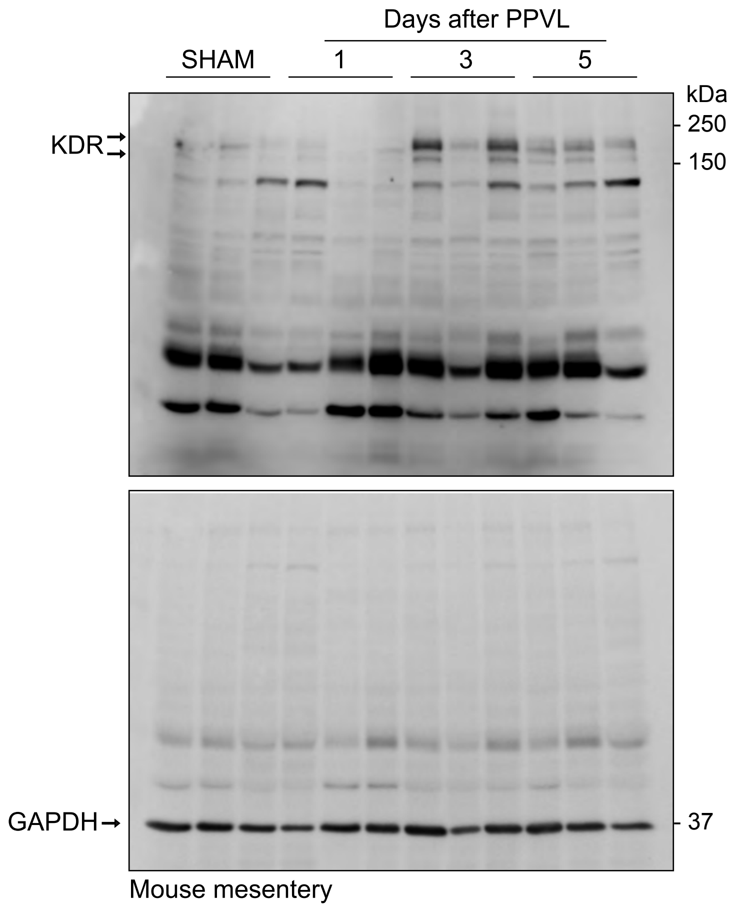

C Full blots corresponding to Figure 5D

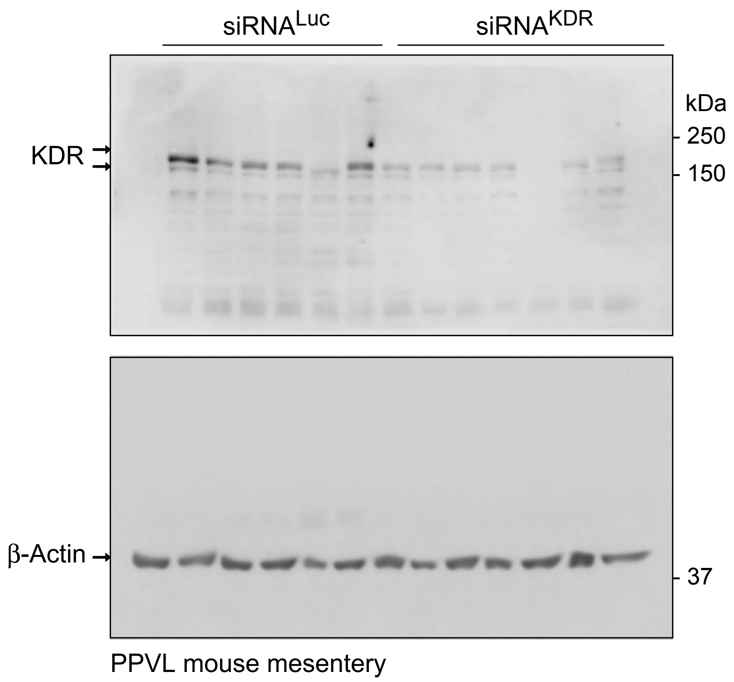

D Full blots corresponding to Figure 6C

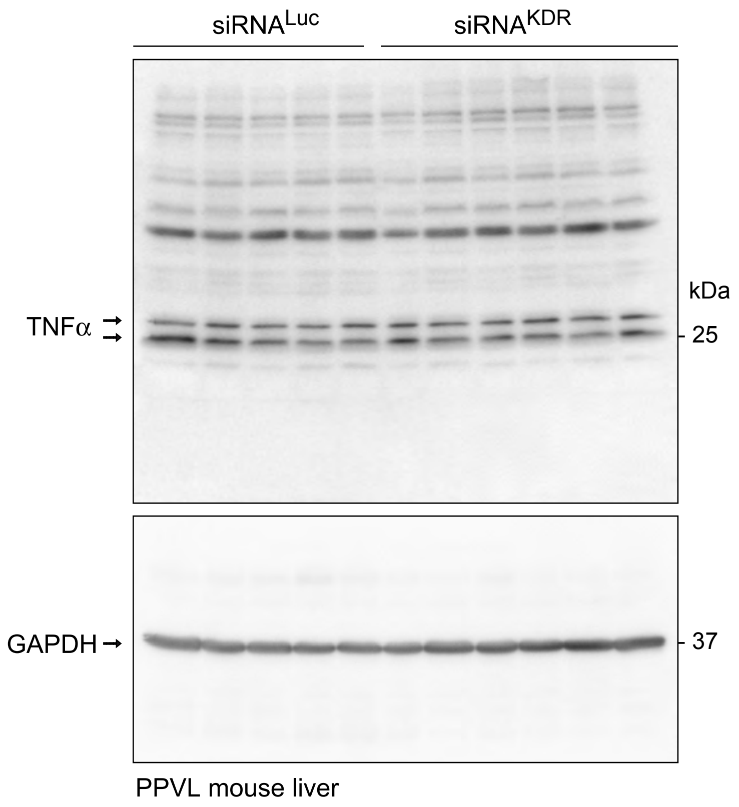

A

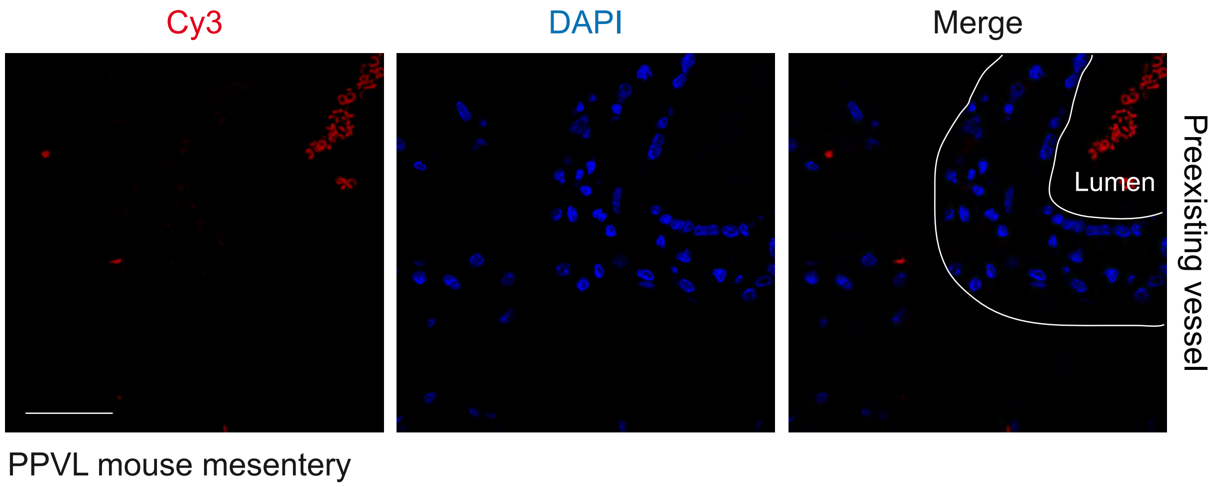
Cy3
DAPI
Merge
Lumen
Preexisting vessel
PPVL mouse mesentery

B

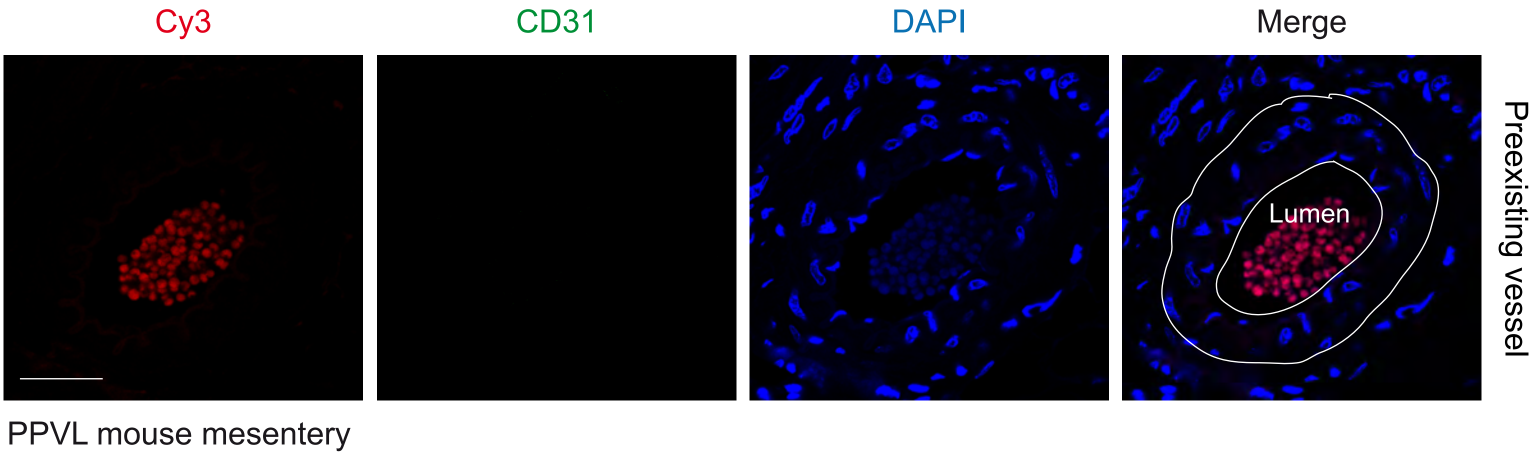
Cy3
CD31
DAPI
Merge
Lumen
Preexisting vessel
PPVL mouse mesentery

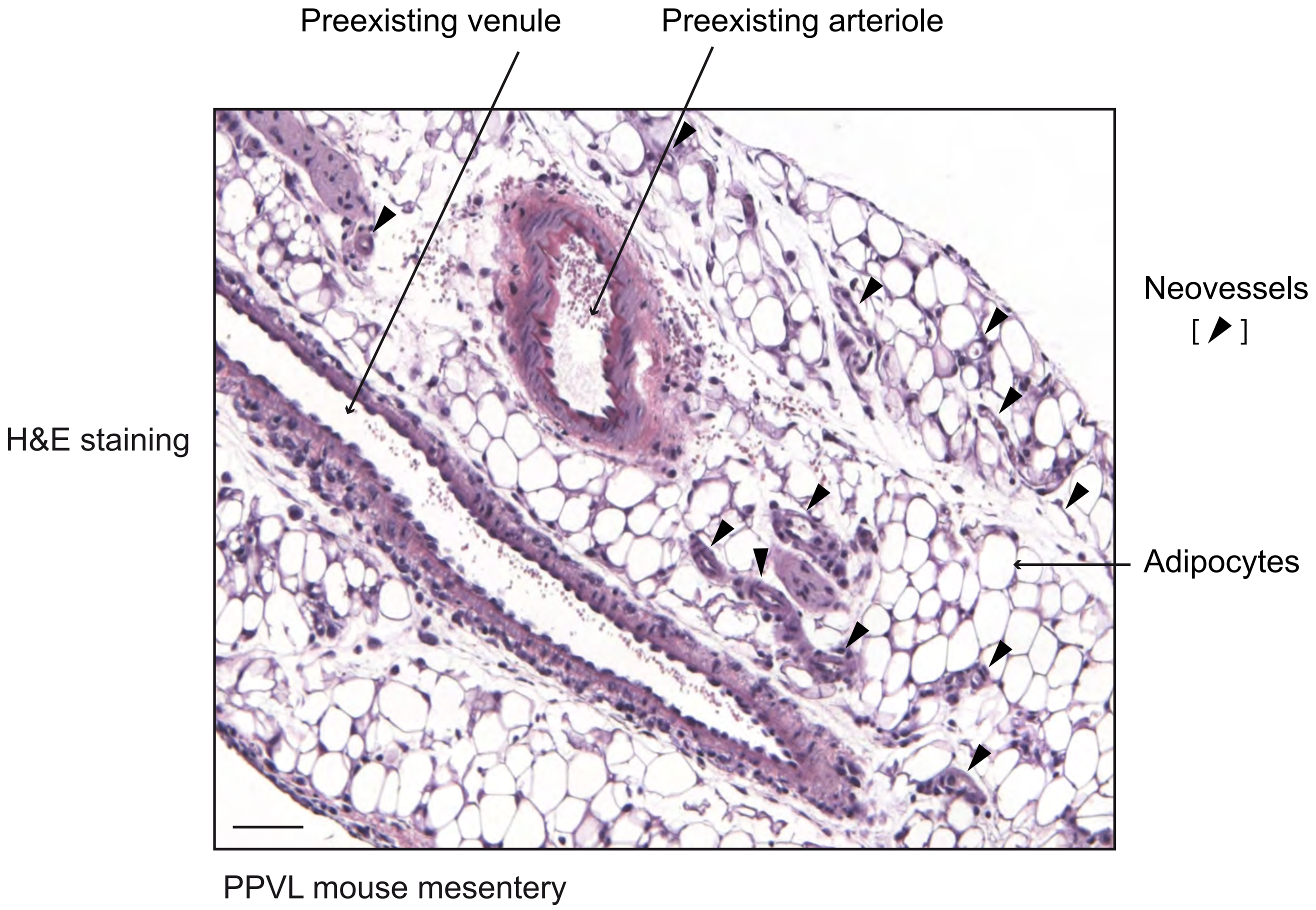
Preexisting venule
Preexisting arteriole
Neovessels
[▲]
H&E staining
Adipocytes
PPVL mouse mesentery

A Full blots corresponding to Figure 7C

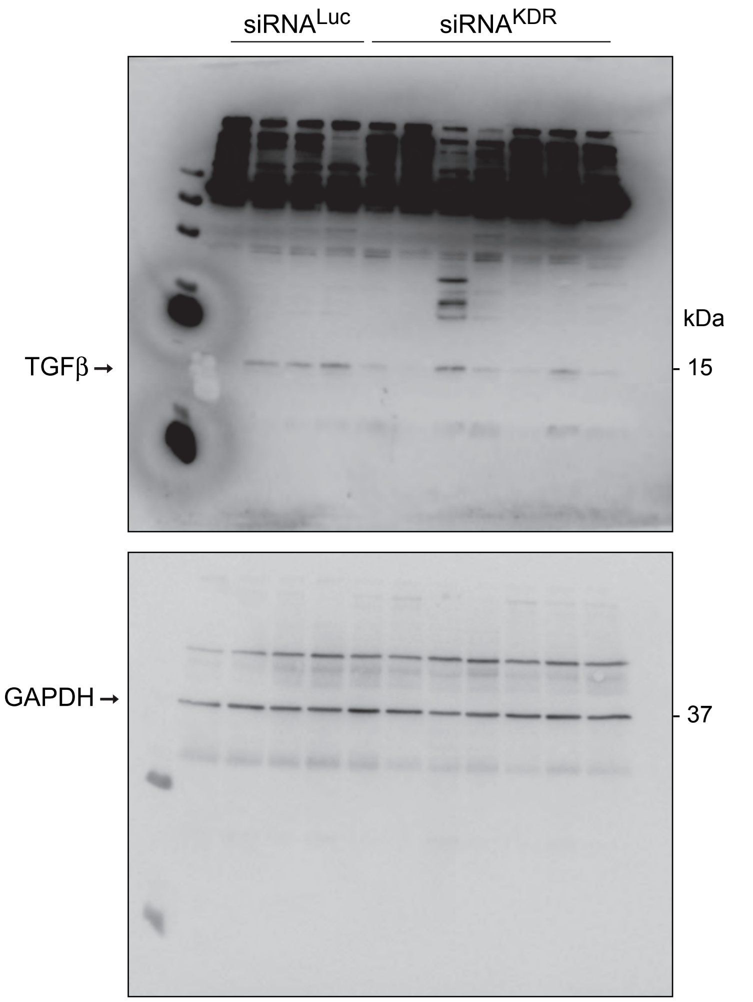

PPVL mouse portal vein

B Full blots corresponding to Figure 7D

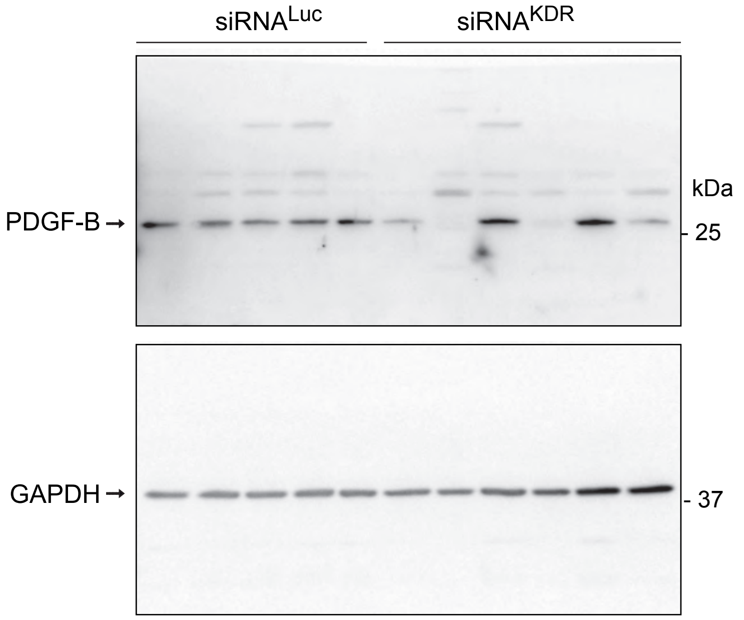

PPVL mouse portal vein

C Full blots corresponding to Figure 8C

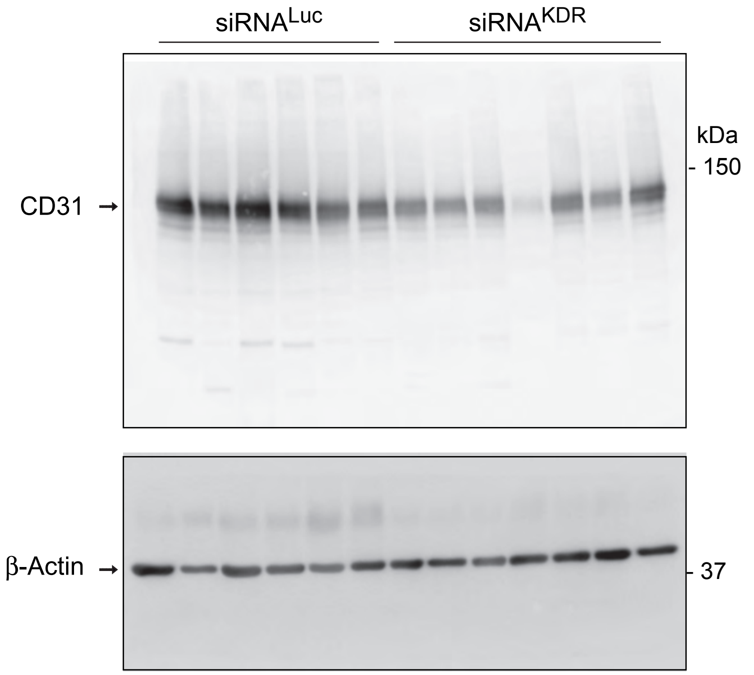

PPVL mouse mesentery

D Full blots corresponding to Figure 8G

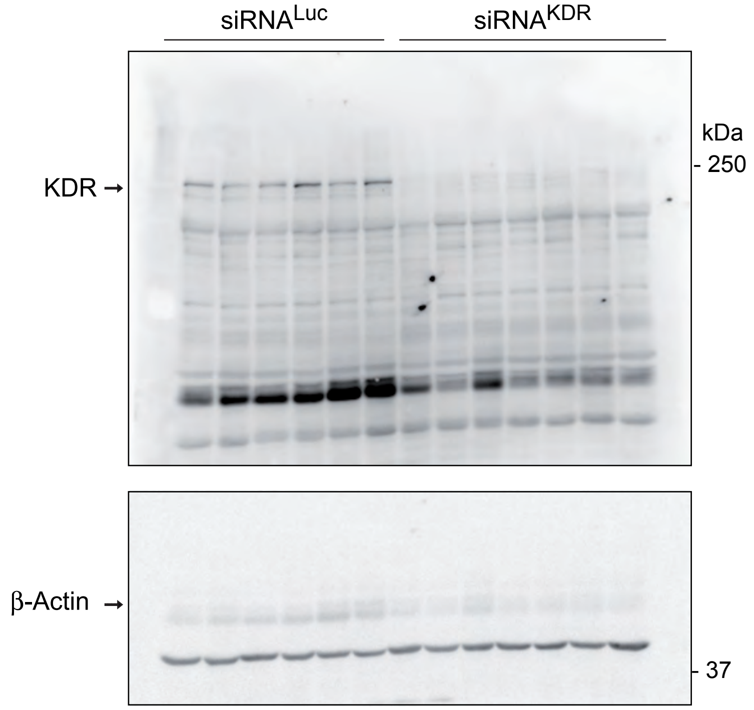

PPVL mouse liver
